# Employment relations and dismissal regulations: Does employment legislation protect the health of workers?

**DOI:** 10.1111/spol.12487

**Published:** 2019-02-14

**Authors:** Pepita Barlow, Aaron Reeves, Martin McKee, David Stuckler

**Affiliations:** ^1^ Bennett Institute for Public Policy, Department of Politics and International Studies University of Cambridge Cambridge UK; ^2^ International Inequalities Institute London School of Economics and Political Science London UK; ^3^ Department of Social Policy and Intervention University of Oxford Oxford UK; ^4^ Faculty of Public Health and Policy London School of Hygiene and Tropical Medicine London UK; ^5^ Carlo F. Dondena Centre for Research on Social Dynamics and Public Policy, Department of Social and Political Sciences Bocconi University Milan Italy

**Keywords:** dismissal legislation, health, insecurity, institutions, job loss, precariousness

## Abstract

Sociologists have long acknowledged that being in a precarious labour market position, whether employed or unemployed, can harm peoples' health. However, scholars have yet to fully investigate the possible contextual, institutional determinants of this relationship. Two institutions that were overlooked in previous empirical studies are the regulations that set minimum compensation for dismissal, severance payments, and entitlements to a period of notice before dismissal, notice periods. These institutions may be important for workers' health as they influence the degree of insecurity that workers are exposed to. Here, we test this hypothesis by examining whether longer notice periods and greater severance payments protect the health of labour market participants, both employed and unemployed. We constructed two cohorts of panel data before and during the European recession using data from 22 countries in the European Union Statistics on Income and Living Conditions (person years = 338,000). We find more generous severance payments significantly reduce the probability that labour market participants, especially the unemployed, will experience declines in self‐reported health, with a slightly weaker relationship for longer notice periods.

## 1 INTRODUCTION

Sociologists have long acknowledged the importance of employment in determining peoples' health. People who lose work experience increased precariousness and suffer worse physical and mental health (Paul & Moser, 2009). But, employment alone is no guarantee of good health. Work, too, can be insecure or precarious and can harm both mental and physical health (Benach & Muntaner, 2007b). Importantly, the risk of these negative health consequences is increasing in an era of rising precariousness (International Labour Organization, 2015). In Europe, 59% of workers were employed on a full‐time, permanent basis in 2014, and the European Commission expects this proportion to decline further (European Commission, 2016). This follows a series of changes to the organization of work in Europe since the 1970s, such as de‐unionization and de‐regulation (Kalleberg, 2009). At the same time, the protections extended to labour market participants became increasingly differentiated or “dualized,” with greater protections for securely employed “insiders” compared with insecurely employed and unemployed “outsiders” (Emmenegger, Hausermann, Palier, & Seeleib‐Kaiser, 2012).

Cross‐national research has investigated how a society's institutions may be important for understanding the determinants of labour market participants' health and what policies could protect workers from the risks associated with job loss and insecurity. Scholars predominantly analysed the effects of welfare regimes and associated labour market policies (Bambra & Eikemo, 2009; Sjoberg, 2010; Stuckler, Basu, Suhrcke, Coutts, & McKee, 2009; Vuori & Silvonen, 2005). However, it is plausible that labour market participants' health is influenced by additional institutions, including legal rules to a period of notice before dismissal (notice periods) and compensation for dismissal (severance payments). Here, we test the hypothesis that legislation controlling minimum notice periods and severance payments protects the health of labour market participants and whether the impact of these rules varies in different macroeconomic contexts.

## 2 BACKGROUND

### 2.1 Employment status and health

Work links individuals to each other, locates people within the stratification system, and is central to individual identity (Kalleberg, 2009). Whether individuals work therefore affects peoples' health and well‐being (Marmot, 2005). People who are not employed and who want to find work are considerably more likely to get sick, have worse mental health, have suicidal thoughts, and die prematurely compared with their employed counterparts (Garcy & Vagero, 2012; Paul & Moser, 2009). These effects can be mediated by a range of material, behavioural, and psychosocial mechanisms (Domenighetti, D'Avanzo, & Bisig, 2000; Ferrie, Shipley, Stansfeld, & Marmot, 2002; Ferrie, Westerlund, Virtanen, Vahtera, & Kivimki, 2008). One is financial strain, which can undermine the ability to satisfy material and social needs and create feelings of low personal control.

Yet, employment alone is no guarantee of good health as some forms of employment can be harmful too. Insecure work, for example, can increase the likelihood of poor mental health and premature mortality (Ferrie et al., 2002). Many mechanisms linking unemployment to poor health can also apply to employed persons. For example, job insecurity can generate concern about future financial security and create feelings of low personal control. Like unemployment, job insecurity, financial strain, and stress are detrimental to physical and mental health and can precipitate behavioural responses that cause further harm, such as excess alcohol consumption.

What is common to job loss and insecurity is precariousness: a multifaceted concept referring to risky, uncertain, and unpredictable labour market positions (Standing, 1997). An extensive empirical literature documents the inimical consequences of both job insecurity and unemployment for peoples' health. For example, persons in temporary, part time, or contingent employment are twice as likely to report worse health (Kim, Kim, Park, & Kawachi, 2008) and 42% more likely to suffer from fatigue than those in stable employment (Benavides, Benach, Diez‐Roux, & Roman, 2000). Although job loss and insecurity may be both consequential and causal in relation to poor health, longitudinal studies of these relationships identify an association running from job loss and job insecurity to poor health independent of baseline health status (Daly & Delaney, 2013; Olesen, Peter Butterworth, & Pirkis, 2013; Strandh & Nordlund, 2008; Virtanen, Janlert, & Hammarström, 2011).

### 2.2 Institutions and workers' health

Institutional theories of the social determinants of health emphasize how the social causes of illness—including insecurity and job loss—can, in turn, be shaped by a society's social, political, and economic institutions (Beckfield et al., 2015). One such institution is a society's welfare regime, which vary in the degree to which they “de‐commodify” workers as “individuals or families can uphold a socially acceptable standard of living” independently of market participation (Esping‐Anderson, 1990, p. 37). Countries are usually classified into welfare regimes according to their scores on an index capturing this de‐commodification via the provision of pensions, unemployment insurance, and sick pay (Bambra & Eikemo, 2009). Studies investigating the institutional determinants of the relationship between employment and health have therefore investigated how welfare regimes and the policies they encompass protect the health status of employed and unemployed labour market participants by reducing exposure to precariousness and financial insecurity (Cylus, Glymour, & Avendano, 2015; Sjoberg, 2010; Stuckler et al., 2009; Vuori & Silvonen, 2005).

Yet, the social determinants of labour market participants' health may be structured by additional institutions that were not incorporated into previous welfare regime and policy analyses. Institutional theories of precariousness call attention to these additional arrangements by highlighting how a much broader range of institutions structure exposure to precariousness (Appelbaum, 2012; Kalleberg, 2009; Kalleberg, Reskin, & Hudson, 2000). There are many such institutions, all of which may vary in generosity between countries and according to an employee's contract or status. They include, for example, employment legislation, “flexicurity” models, which combine minimal labour market regulations with social security, and unionization regulations. According to Kalleberg, what is central to the possible influence of these institutions is whether they re‐calibrate “employment relations”: the relationships between employees and employers that vary in their relative power to set the conditions of employment and dismissal (Kalleberg, 2009). The institutions that re‐calibrate asymmetric relationships in setting dismissal conditions could be especially important in protecting labour market participants' health as they protect workers from the feelings of insecurity and financial strain associated with job loss.

### 2.3 Dismissal legislation and health

Legislation controlling dismissal procedures is designed to re‐calibrate power asymmetries in setting dismissal conditions and, by doing so, may have a beneficial impact on the health of workers and the unemployed. Multiple regulations control dismissal, as set out in a suite of laws known as “employment protection legislation” (OECD, 2014) and may impact labour market participants' health through various mechanisms. Two rules that may be especially important are those that set workers' minimum entitlements to a period of notice before dismissal (“notice periods”) and minimum compensation for dismissal (“severance payments”). These two rules are designed to protect workers from an employer's potentially unencumbered power to dismiss them without sufficient warning and compensation (Deakin, Lele, & Siems, 2007). Such rules may be particularly important because financial strain and fearing dismissal are important mediators of the relationships between insecurity, job loss, and health (Huijts, Reeves, McKee, & Stuckler, 2015; Meltzer et al., 2010) and because theories of precariousness stress the importance of institutions that re‐calibrate dismissal conditions (Kalleberg et al., 2000).

Legislation requiring higher severance payments may protect health among the employed and unemployed via a “financial strain mechanism.” For the employed, greater severance payments could reduce the possibility and fear of income loss and associated financial strain upon dismissal. For the unemployed, greater severance payments could directly protect against financial strain and its harms by reducing the decline in income when transitioning from employment to unemployment (Huijts et al., 2015). Higher severance payments may also provide the resources necessary to develop new interests, skills, and activities, creating a renewed sense of autonomy and competence or providing resources for a career change, leading health to stabilize or recover (Atkinson, 2010; Warr, 1987).

Legislation requiring longer notice periods could also reduce precariousness and protect health among the employed and unemployed via an “employment shock mechanism.” For the employed, longer notice periods could minimize feelings of insecurity by legally precluding the possibility and fear of rapidly becoming unemployed and associated stresses. For the unemployed, longer notice periods can provide individuals with a longer period to adjust and adapt to the shock of losing their jobs whilst still in work. Adaptation might be financial, by providing a longer saving period to prepare for the loss of future income; psychological, with a longer period to adjust to the actual shift in employment status from employed to unemployed; and occupational, providing a period to plan an alternative career or activity.

The protective effects of severance payment and notice period legislation are likely to be stronger among the unemployed who are in a more vulnerable position—and so more reliant on the legislation—having lost their jobs. In our analysis, we therefore examine this possible heterogeneity. There may also be variation among employed persons: Those who are temporarily employed and so are labour market “outsiders” may not benefit from the legislation to the same extent as securely employed persons (“insiders”) as the former may be ineligible for the regulations (Emmenegger et al., 2012). Unfortunately, we were unable to fully examine this source of heterogeneity due to the insufficient numbers of temporarily employed persons in the panel component of the European Union Statistics on Income and Living Conditions (EU‐SILC).

### 2.4 Dismissal legislation during recessions

The effectiveness of dismissal legislation may also be attenuated during macroeconomic conditions. Recession may associate with reduced compliance with the legislation, removing its protective effects (Reeves, Karanikolos, Mackenbach, McKee, & Stuckler, 2014). They can also increase labour market participants' exposure to stress due to stagnating wages, in addition to rising fear or risk of long‐term unemployment (Drydakis, 2015). In addition, many European governments responded to the 2008–09 recession by reducing spending on unemployment assistance, mental health services, and other social programmes, potentially exacerbating the detrimental health effects of job loss and worsening workers' well‐being (Reeves, McKee, Basu, & Stuckler, 2014). These changes may have eroded the protective effect of dismissal legislation because it was not an adequate buffer in a context of rising insecurity and without other sources of social and health services support.

### 2.5 Literature

Previous scholarship on institutions and labour market participants' health predominantly analysed welfare regimes and the policies they compass, or those that function outside the workplace after a worker is dismissed. For example, an extensive literature has examined the protective effects of welfare regimes, identifying a positive association between the generosity of welfare regimes and labour market participants' health, both employed and unemployed (Bambra & Eikemo, 2009; Kim et al., 2012). A related literature analysed specific policies, including the role of cash benefits, which provide material assistance for the unemployed and so reduce reliance on labour market income, as well as job search programmes, which help workers re‐train and re‐enter the labour market (Cylus et al., 2015; Sjoberg, 2010; Stuckler et al., 2009; Vuori & Silvonen, 2005). Although others have highlighted the possible importance of dismissal legislation in protecting labour market participants' health, to our knowledge, no previous studies have empirically tested this hypothesis (Barlow, Reeves, McKee, & Stuckler, 2015; Benach & Muntaner, 2007a; Kim et al., 2008; Muntaner et al., 2011).

Here, we test the hypothesis that legislation requiring greater severance payments and longer notice periods protect the health of labour market participants (Hypothesis 1) and whether this association varies by employment status (Hypothesis 2). We also test whether rising insecurity, stagnating incomes, and lower prospects of re‐employment associated with macroeconomic recession weakens the protective effects of notice periods and severance payments (Hypothesis 3). Finally, we test whether the associations between dismissal legislation and health are mediated by reductions in financial strain (Hypothesis 4).

## 3 METHODS

### 3.1 Data and measurement

We assembled individual‐level panel data from the EU‐SILC, covering 22 EU and OECD member countries. These were matched with aggregate data on Gross Domestic Product (GDP), unemployment rates (% of the labour force), and public expenditure on the unemployed from Eurostat (2015) and the OECD General Statistics and Unemployment databases (European Commission, 2015; OECD, 2015). The details of the EU‐SILC have been described elsewhere (Arora et al., 2015); however, briefly, it uses a rotational design, replacing 25% of the sample each year with a maximum coverage of 4 years. We constructed two cohorts from the longitudinal component of the EU‐SILC. The first cohort uses the 2007 release of the survey and covers a period before the recession, 2005–07. The second cohort uses the 2010 release of the survey and covers a period during the recession, 2008–10; 2008 is the first recession year because GDP declines began in late 2007 and early 2008 (European Commission, 2015). In response to the recession, many European countries reduced social spending from 2010 onwards (Reeves, McKee, et al., 2014), so we chose 2010 as the cut‐off point to distinguish the effects of the recession from subsequent policy changes.

Following existing studies and recommendations in Allison (1990), we measured health status using a dichotomous indicator for whether self‐reported health (SRH) status declined between two survey waves (Barlow et al., 2015; Ferrarini, Nelson, & Sjöberg, 2014). SRH is a widely used health measure that is highly correlated with mental and physical health (Jylha, 2009) and even may be a better mortality predictor than some objective measures such as a long‐standing illness diagnosis (Ganna and Ingelsson, 2015). We model change in SRH over time as the cross‐sectional association with its level could capture the influence of unobserved, time‐invariant factors that influence health in the base year and subsequent health outcomes. Modelling change over time addresses this potential source of bias (Allison, 1990; Angrist & Pischke, 2009).

Severance payments and notice periods are our key explanatory variables, measured using the OECD Employment Protection Legislation Index (OECD, 2014). The full index captures 21 labour market regulations. We confine our analysis to the two main regulations related to dismissal that are most likely to directly affect the lives of workers via changes to dismissal conditions: severance payment and notice period legislation. To construct the index, the OECD codes raw data on notice period and severance pay entitlements into seven scores ranging from 0 to 6, with higher scores reflecting higher severance pay or longer notice periods. The scores are separated according to three different employment durations as the entitlements usually vary by an employees' tenure. The OECD then sums the scores across the three tenures, creating a score that ranges from 0 (*least generous*) to 18 (*most generous*; see Supporting Information 1). We group countries into four quartiles based on their severance payment and notice period scores in each year, aiming at ~25% of observations in each quartile, with higher quartiles representing greater protection. There is very little variation in the full OECD index within countries in our study period, so the raw scores and their conversion into quartiles is constant over time in most countries (see Supporting Information 2).

Grouping countries into quartiles addresses two challenges. First, some scores on the full OECD index are occupied by only a single country, whereas other scores are occupied by no countries. Estimating a model using the raw score therefore lacks validity on some values of the scale and would create difficulties in disentangling country scores from potential confounders, such as welfare regime type (Bambra, 2007). Second, the raw OECD index is not intuitively interpretable as scores are assigned based on dismissal legislation at three different tenure lengths. Grouping countries into quartiles addresses these problems by facilitating intuitive interpretation and overcoming the sparse data problem, enabling us to adjust for covariates. This coding decision did not affect our findings (see Supporting Information 3). Importantly, the Scandinavian countries belong to both high and low‐severance payment quartiles, obviating the possibility that our results are attributable to the protective effects of the more generous welfare regimes in Scandinavian countries.

We test whether these relationships are also associated with financial strain, which is one potential mediator of the relationship between dismissal legislation and health (Kalleberg, 2009). We constructed a binary measure of whether individuals reported difficulties in making ends meet, responding “with some difficulty,” “with difficulty,” or “with great difficulty” when asked whether their household is able to “pay for its usual necessary expenses.” We then calculate changes in this variable between survey waves to construct a dichotomous indicator of whether individuals fell into difficulties (1) or not (0).

### 3.2 Sample

Following existing studies of labour market change, we excluded inactive persons such as those who were retired, studying, or younger than 18 or over 64. To account for potential “healthy worker effects,” that is, those in poor health are more likely to become unemployed (Bartley, 1994), we restricted our sample to individuals who were employed in the first year of each cohort (2005 and 2008). In the second year of each cohort (2006 and 2009), we then included persons who had been unemployed, economically inactive or not interviewed in the first year (2005 and 2008) but were now employed. Finally, we excluded individuals who were missing data on our key explanatory variables. To check whether this could bias our results, we conducted tests of dependence between missing data on each variable and the probability of a health decline (Supporting Information 4). Those with missing education data were more likely to report a health decline (Test of independence: χ^2^(1) = 22.5, *p* < 0.001) suggesting that their exclusion created a potential downward bias in our hypothesis testing.

Table 1 displays descriptive statistics for individual data, and Table 2 displays descriptive statistics for country‐level data. Our final analytic sample comprised 348,000 person years, including 175,326 observations from 2005 to 2007 (107,937 respondents) and 172,674 observations from 2008 to 2010 (108,813 respondents). The proportion of individuals who became unemployed between survey waves varied between 2.4% (2006–07) and 3.7% (2010–11). The mean severance payment quartile varied between 2.1 (2005–06) and 2.5 (2009–10), and the mean notice period quartile varied from 2.4 (2005–06) to 2.6 (2008–09).

**Table 1:** Descriptive statistics: individual‐level variables, 2005–2010

	Prerecession cohort				Recession cohort			
Variable	2005–2006		2006–2007		2008–2009		2010–2011	
	Mean or %	N	Mean or %	N	Mean or %	N	Mean or %	N
Health decline	19.76%	14,428	18.67%	19,104	18.06%	13,153	17.38%	17,347
	(0.40)		(0.39)		(0.38)		(0.38)	
Employment								
Remain	97.30%	71,043	97.64%	99,896	96.40%	70,218	96.26%	96,101
Employed	(0.16)		(0.15)		(0.2)		(0.19)	
Become	2.70%	1,971	2.36%	2,416	3.60%	2,622	3.74%	3,733
Unemployed	(0.16)		(0.15)		(0.20)		(0.19)	
Age^γ^	42	73,014	42	102,312	43	72,840	43	99,834
	(10.77)		(10.81)		(10.8)		(10.8)	
Sex								
Male	55.31%	40,384	54.95%	56,225	53.85%	39,225	53.48%	53,395
	(0.50)		(0.50)		(0.50)		(0.50)	
Female	44.69%	32,630	45.05%	46,087	46.15%	33,615	46.52%	46,439
	(0.50)		(0.50)		(0.5)		(0.50)	
Marital Status								
Married	62.43%	45,583	62.28%	63,719	60.74%	44,241	60.69%	60,594
	(0.48)		(0.48)		(0.49)		(0.49)	
Not married	37.57%	27,431	37.72%	38,593	39.26%	28,599	39.31%	39,240
	(0.48)		(0.48)		(0.49)		(0.49)	
Education								
Pre (primary and primary	9.54%	6,962	8.51%	8,704	7.78%	5,666	7.50%	7,485
	(0.49)		(0.28)		(0.26)		(0.26)	
Lower secondary	14.45%	10,553	14.08%	14,408	14.74%	10,736	14.89%	14,862
	(0.49)		(0.35)		(0.35)		(0.36)	
Upper secondary	46.51%	33,959	47.68%	48,783	45.98%	33,494	45.98%	45,908
	(0.50)		(0.50)		(0.50)		(0.50)	
Post (secondary	4.29%	3,132	4.52%	4,621	3.34%	2,435	3.08%	3,079
	(0.43)		(0.21)		(0.18)		(0.17)	
Tertiary	25.21%	18,408	25.21%	25,796	28.16%	20,509	28.55%	28,500
	(0.40)		(0.43)		(0.45)		(0.45)	
Occupation								
Legislators, senior officials, and managers	9.19%	6,710	8.90%	9,106	8.77%	6,388	8.70%	8,686
	(0.10)		(0.14)		(0.26)		(0.26)	
Professionals	14.62%	10,675	14.45%	14,784	15.88%	11,567	16.04%	16,013
	(0.35)		(0.31)		(0.37)		(0.37)	
Technical and assoc. professionals	16.40%	11,974	16.31%	16,687	17.37%	12,652	17.42%	17,391
	(0.37)		(0.33)		(0.31)		(0.38)	
Other assoc. professionals	10.73%	7,834	10.70%	10,947	10.74%	7,823	10.56%	10,542
	(0.31)		(0.21)		(0.33)		(0.31)	
Clerks	12.60%	9,200	12.68%	12,973	12.41%	9,039	12.75%	12,729
	(0.33)		(0.33)		(0.21)		(0.33)	
Service, shop, and sales workers	4.62%	3,373	4.40%	4,502	4.39%	3,198	4.55%	4,542
	(0.21)		(0.21)		(0.34)		(0.21)	
Craft and trades workers	14.60%	10,660	14.91%	15,255	13.38%	9,746	13.07%	13,048
	(0.35)		(0.36)		(0.28)		(0.33)	
Plant and machine operators	9.04%	6,600	9.20%	9,413	8.84%	6,439	8.50%	8,486
	(0.29)		(0.29)		(0.28)		(0.28)	
Elementary occupations	8.20%	5,987	8.45%	8,645	8.22%	5,987	8.41%	8,396
	(0.27)		(0.28)		(0.28)		(0.27)	
Total in each year		73,014		102,312		72,840		**99,834**

*Notes*: γ: age range = 18 to 65 years in all periods. All other variables take a value of 0–1 in each category. Figures show the proportion of cases in each category, except age, which measures the mean age. Standard deviations in parentheses.

**Table 2:** Descriptive statistics: country‐level variables, 2005–2010

Variable	Description	Pre‐recession cohort		Recession cohort	
		2005–2006	2006–2007	2008–2009	2009–2010
Severance payment quartile	Measure of legally mandated minimum severance payment upon dismissal	2.14 (0–3; 1.10)	2.17 (0–3; 1.17)	2.41 (0–3; 1.13)	2.49 (0–3; 1.09)
Notice period quartile	Measure of legally mandated minimum period of notice	2.4 (0–3; 1.09)	2.43 (0–3; 1.09)	2.66 (0–3; 0.99)	2.54 (0–3; 1.03)
GDP	Per capita, in US $, constant prices, constant PPS, reference year 2005	30,075.37 (14,674.71‐69,291.81;11,630.22)	30,185.02 (15,738.16‐72,573.24; 11,163.91)	29,341.14 (16,1149.96‐66,612.18; 10,858.94)	29,025.22 (15,579.66‐68,755.69; 10,544.43)
Change in GDP	Percentage change in GDP	3.74% (1.33–11.08; 2.23)	3.57% (0.4–10.57; 2.76)	−4.65% (−14.57–2.53; 3.04)	1.28% (−5.16–5.09; 1.99)
Unemployment rate	% economically active population in employment	7.76 (2.88–13.97; 2.94)	6.74 (2.29–11.23; 2.13)	8.96 (3.15–17.9; 3.68)	10.13 (3.58–19.9; 4.35)
Change in unemployment	Percentage change in unemployment	−8.57% (−25.50 11.65; 9.62)	−12.32% (−31.21–3.88; 9.76)	31.90% (4.06–146.07; 26.72)	10.13% (−10.42–32.44; 9.77)
Unemployment spending	Per capita, in Euros. Transfers, in cash or in kind to households and individuals to relieve them of the risks or needs associated with unemployment	114.69 (4–512.23; 128.25)	116.66 (15–514.56; 131.71)	150.52 (13–580.84; 233.06)	149.44 (7–780.85; 219.33)

*Notes*. Minimum, maximum, and standard deviations in parentheses (min–max; standard deviation). Unemployment spending data from Eurostat, all other data are taken from OECD. GDP: gross domestic product.

### 3.3 Modelling

Following previous studies of labour market institutions and health, we estimate multilevel logistic regression models per Equation 1 (Reeves, Karanikolos, et al., 2014):

$$\text{Equation}\;1:{\text{Health decline}}_{\mathrm{ijt}}=\;\beta_{0}+\beta_{1}\;{\text{Dismissal legislation}}_{\mathrm{jt}}+\beta_{2}\mathrm{Job}\;{\text{loss}}_{\mathrm{ijt}}+\beta_{3}\;{\mathrm{Age}}_{\;\mathrm{ijt}}+\beta_{4}\;{{\mathrm{Age}}^{2}}_{\mathrm{ijt}}+\beta_{5}\;{\text{Female}}_{\mathrm{ijt}}+\beta_{6}\;{\text{Education}}_{\mathrm{ijt}}+\beta_{7}\;{\text{Married}}_{\mathrm{ijt}}\;+\beta_{8}\;{\text{Employment sector}}_{\mathrm{ijt}}+\beta_{9}\;{\mathrm{GDP}}_{\mathrm{jt}}+\beta_{10}\;{\mathrm{∆GDP}}_{\mathrm{jt}}+\beta_{11}\;{\mathrm{UE}}_{\mathrm{jt}}+\beta_{12}\;{\mathrm{∆UE}}_{\mathrm{jt}}+\beta_{13}{\mathrm{US}}_{\mathrm{jt}}+\varepsilon_{\mathrm{itj}}.$$

Here, *i* is individual, located in country *j*, in year *t*. Separate models were estimated for each type of employment protection and in each period before (2005–07) and during the recession (2008–10). *Health decline* is the probability of experiencing a decline in self‐reported health between two survey waves. *Dismissal legislation* is either severance period quartile (1 to 4) or notice period quartile (1 to 4) in which countries lie at time *t*. *β*_1_ is the estimate of the effect of this dismissal legislation on the probability of a health decline. *Job loss* is the measure of becoming unemployed, based on a self‐defined measure of current economic status.

To adjust for potentially confounding variables of the associations with health, we incorporate controls for age (Artazcoz, Benach, Borrell, & Cortès, 2004), sex (Backhans & Hemmingsson, 2012), marital status (Howe, Levy, & Caplan, 2004), sector of employment (Creek & Hughes, 2008), and education level (Turner, 1995). *Age* is age at the second survey wave. *Age* ^*2*^ adjusts for a non‐linear association between age and the probability of a health decline. *Female* is 1 if female, 0 male. *Education* is a series of dummy variables, re‐coding highest education level achieved into four categories: “pre‐primary and primary” (baseline), “lower secondary,” “upper secondary,” and “tertiary” education. *Married* is a dummy variable capturing whether individuals were married or cohabiting, and *Employment sector* is coded into the nine major groupings of the International Standard Classification of Occupations (ISCO‐88; European Commission, 2018). To account for bias in the standard errors due to correlations within individuals over time, we clustered the standard errors at the individual level.

We also adjust for macroeconomic variables, which may moderate the association between severance payments, notice periods, employment status, and health. *GDP* is gross domestic product per capita, adjusted for purchasing power and measured in constant prices (2005 US dollars); *UE* is the unemployment rate, and ΔGDP and ΔUE measure changes in GDP and unemployment (Catalano et al., 2011; Chadi, 2014; Economou, Nikolaou, & Theodossiou, 2008). *US* is spending on unemployment benefits per capita, in Euros (Ferrarini et al., 2014).

Our data are hierarchical and so we estimate multilevel logistic regression models. We do so with a largely time‐invariant country‐level legislation index variable due to the lack of variation in the employment legislation index over time. This prevents estimation of a country‐level fixed‐effects model (due to insufficient within‐country variation) and also prevents us from estimating country‐level effects with standard multilevel models (Bryan & Jenkins, 2016).

We first estimate our model as per Equation 1. Then, we test whether dismissal legislation changes the relationship between employment status and health by re‐estimating Equation 1 with a *dismissal legislation × job loss* interaction. Again, *dismissal legislation* is either severance period quartile (1 to 4) or notice period quartile (1 to 4). We then compute marginal effects based on specified parameter values using STATA's margins command (Norton, 2004; Williams, 2012). To test for variation according to macroeconomic conditions, we also estimate Equation (1) and the interaction model separately for the prerecession panel and the recession panel.

We test whether these relationships are also associated with financial strain following Baron and Kenny's procedure for testing mediation and examine four relationships: Step 1 dismissal legislation and health, Step 2 financial strain and health, Step 3 dismissal legislation and financial strain, and Step 4 health and dismissal legislation, adjusting for financial strain (Baron & Kenny, 1986). The goal of the first three steps is to establish whether relationships between the explanatory variable, outcome variable, and mediator actually exist; the goal of the final step is to evaluate whether financial strain fully or partially mediates the association in Equation 1 (see Supporting Information 5 for additional detail). We again model change in financial strain over time for consistency with our main analysis and to adjust for unobserved (time invariant) confounders. Finally, we performed a series of robustness tests for our sample and model specification. All models were estimated in STATA 13.

## 4 RESULTS

### 4.1 Does dismissal legislation protect labour market participants' health?

We first evaluated Hypothesis 1, examining the association between health and different severance payments and notice periods before the recession in the years 2005–07 regardless of employment status. The proportion of individuals who reported a decline in self‐reported health between survey waves varied between 15.6% (Sweden) and 24.5% (Greece). Table 3 reports the results of our multilevel models and shows that across all groups, both employed and unemployed, each quartile increase in severance payments was associated with a 7% lower relative odds of reporting a health decline before the recession (Adjusted Odds Ratio [AOR]: 0.93, 95% CI [0.91–0.94]; Table 3; see Supporting Information 6–8 for full models). To put the magnitude of these associations in perspective, in the Czech Republic (fourth quartile) where workers are entitled to three times their average earnings as a severance package if employment lasted at least 2 years, labour market participants experienced a 15.5% predicted probability of a health decline (95% CI [0.15–0.16]; OECD, 2013a). In contrast, in Slovenia, where workers are entitled to just one‐fifth months' pay as a severance package where employment lasted 1–10 years (first quartile), individuals had a 20.0% probability of a health decline (95% CI [0.195–0.204]; OECD, 2013b).

**Table 3:** Coefficients of job loss, severance payment quartile, and notice period quartile, disaggregated by period

	Job Loss	Severance payment quartile	Notice period quartile
Prerecession	1.27***	0.93***	0.95***
	(0.05)	(0.01)	(0.01)
Recession	1.30***	0.96***	0.96***
	(0.04)	(0.01)	(0.01)

*Notes. p* values:

* ** ***

Exponentiated coefficients show association of a 1‐unit increase in the variable in each column upon the odds of reporting a health decline. *N* = 175,326 in prerecession period (2005–2007) and *N* = 172,674 in recession period (2008–2010). See Supporting Information 6–8 for full models.

Similarly, we found notice periods correlated with a lower odds of a health decline across all groups, both employed and unemployed. Each quartile increase in notice periods was associated with a 5% lower relative odds of reporting a health decline (AOR: 0.95, 95% CI [0.94–0.97]; Table 3).

**Figure 1:**
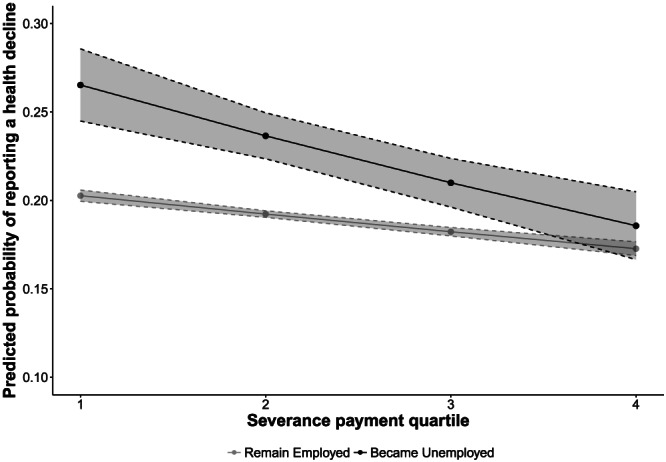
Predicted probability of a health decline by employment status and severance payment quartile before the recession *Notes*. Examples of countries in each quartile: Q1 – Italy, Norway. Q2 – Denmark, Hungary. Q3 – Greece, Slovakia. Q4 – Netherlands, Spain. Predicted probabilities computed after estimating full model with severance payment *x* job loss interaction. See Supporting Information 6–8 for full models.

**Figure 2:**
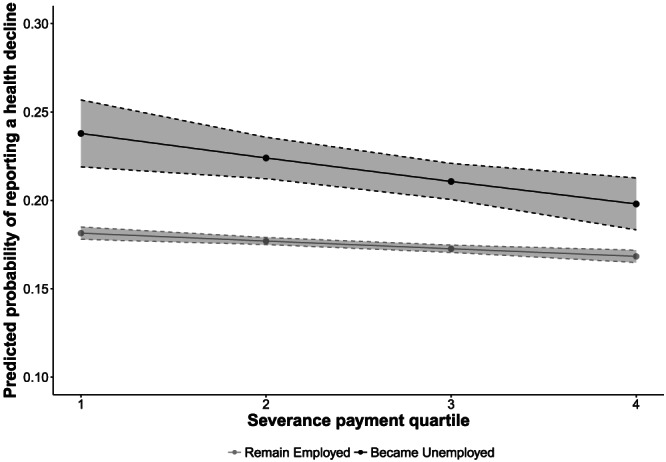
Predicted probability of a health decline by employment status and severance payment quartile during the recession *Notes*. Examples of countries in each quartile: Q1 – Italy, Norway. Q2 – Denmark, Hungary. Q3 – Greece, Slovakia. Q4 – Netherlands, Spain. Predicted probabilities computed after estimating full model with severance payment *x* job loss interaction. See Supporting Information 6–8 for full models.

**Figure 3:**
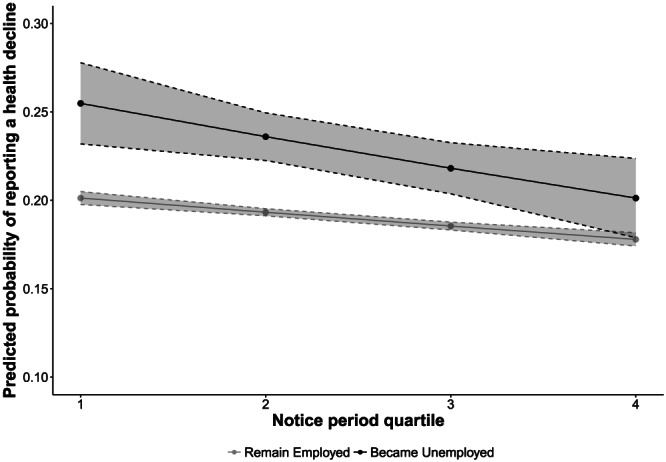
Predicted probability of a health decline by employment status and notice period quartile before the recession *Notes*. Examples of countries in each quartile: Q1 – Austria, Slovenia. Q2 – France, Hungary. Q3 – Greece, Poland. Q4 – Czech Republic, Sweden. Predicted probabilities computed after estimating full model with notice period *x* job loss interaction. See Supporting Information 6–8 for full models.

**Figure 4:**
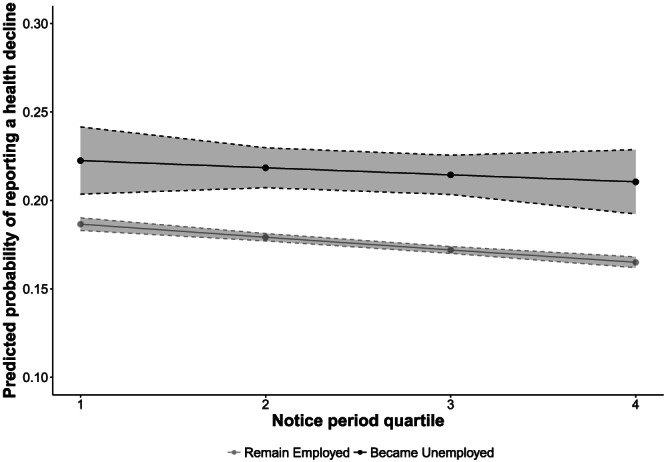
Predicted probability of a health decline by employment status and notice period quartile during the recession *Notes*. Examples of countries in each quartile: Q1 – Austria, Slovenia. Q2 – France, Hungary. Q3 – Greece, Poland. Q4 – Czech Republic, Sweden. Predicted probabilities computed after estimating full model with notice period *x* job loss interaction. See Supporting Information 6–8 for full models.

We then evaluated Hypothesis 2 by disaggregating these associations into those who remained employed and those who lost jobs. Individuals who became unemployed were 1.27 (95% CI [1.37–1.17]) times more likely to report a health decline before the recession and 1.30 (95% CI [1.38–1.22]) times more likely to report a health decline during the recession, compared with those who remained unemployed. As shown in Figures 1, 2, 3, 4, there was a protective association with health for both measures of employment legislation, but associations were stronger for (a) severance payments and (b) in those who became unemployed. Before the recession, those who lost their job in countries with the highest severance payments and notice periods were no more likely to experience a health decline than those who remained employed.

On average, those who became unemployed in the highest severance payment quartile were 8.0% less likely to experience a health decline than those who became unemployed in the lowest quartile (95% CI [5.0–11.0%]). Similarly, those who became unemployed in the highest notice payment quartile were 5.0% less likely to experience a health decline than those who became unemployed in the lowest quartile (95% CI [1.6–9.0%]).

These results are qualitatively unchanged when we adjust for welfare‐regime type, incorporating a dummy variable at the country level following Bambra and Eikomo's (2009) classification (Supporting Information 9). Compared with welfare regimes, our estimate of the effect of severance payments (OR = 0.93; 95% CI [0.92–0.95]) represents 77% of the estimated effect of belonging to Scandinavian welfare regime compared with the Anglo‐Saxon regime (OR = 0.91; 95% CI [0.87–0.95]), whereas estimate of the effect of notice periods (OR = 0.94, 95% CI [0.93–0.95) was 44% of this effect.

### 4.2 Dismissal legislation during the recession

We then tested Hypothesis 3: whether these associations were also observed in the Great Recession years, 2008–10. Table 3 shows that during the recession (2008–10), higher severance payments remained negatively associated with the probability of reporting a health decline. However, this association was weaker than before the recession (test of effect heterogeneity: χ^2^(1) = 14.97, *p* < 0.001), although those who became unemployed in the highest severance payment quartile were still less likely to report a health decline than those in the lowest quartile (difference in predicted probabilities: 0.04; 95% CI [0.01–0.07]). Similar results were observed for notice periods (Supporting Information 6–8). Again, these results were qualitatively unchanged when we adjust for welfare‐regime type (Supporting Information 9).

### 4.3 The role of perceived financial strain

Finally, we evaluated hypothesis 4, that is, whether financial strain partially mediated the association between severance payments and health (Baron & Kenny, 1986; see Supporting Information 5). We have established that severance payments are correlated with the probability of worsening health (Step 1). Next, in Step 2, we also find that those who fell into difficulties with making ends meet were more likely to experience a health decline (AOR: 1.22, 95% CI [1.16–1.29]) than those who did not (Table 1, Supporting Information 5). We then tested whether the probability of a change in financial strain was reduced by dismissal legislation (Step 3). Both forms of dismissal legislation were negatively associated with falling into difficulties with making ends meet (Table 2, Supporting Information 5). For example, individuals in the highest quartile before the recession were 0.97% (95% CI [−1.56% to −0.37%]) less likely to fall into financial strain than those in the lowest quartile. However, this difference was not significant during the recession (estimated difference: −0.4%, 95% CI [−1.16% to 0.26%]). Finally, we examine whether severance payments and notice periods are correlated with the probability of worsening health after adjusting for financial strain (Step 4). Table 3 in Supporting Information 5 shows that the dismissal legislation coefficients were attenuated in these models. Taken together, these models suggest that changes to financial strain partially mediate the association between severance payments and health.

### 4.4 Robustness checks

We conducted a series of robustness checks of our model and sample specification. First, because logistic regression estimates are affected by unobserved heterogeneity (Mood 2010), we re‐estimated our results using a linear probability model. As with our original results, the coefficients on severance payments and notice periods were negative and significant at the *α* = 0.001 level (Supporting Information 10). In addition, by modelling changes in health over time to control for unobserved time‐invariant factors affecting health status, we omitted a control for baseline health status because this would, by construction, be correlated with the error term (Allison, 1990). We nevertheless tested whether our results were robust to the inclusion of a lagged measure of self‐reported health. The severance payment and notice period coefficients were marginally smaller in these models but remained significant and negatively associated with the probability of a health decline (Supporting Information 11).

Next, we re‐estimated our models including those who were initially unemployed but gained employment between survey waves, coding them in the same category as those who remained employed. The associations between severance payments, notice periods, and the probability of a health decline did not change when we included this group (Supporting Information 12). We also conduct an additional test restricting the sample to permanently employed labour market insiders by excluding temporarily employed outsiders from the sample in order to assess whether their inclusion influenced our results. Supporting Information 13 shows that there is no consistent pattern of change in the severance payment and notice period coefficients when we exclude temporarily employed outsiders, suggesting that their inclusion did not influence our findings.

We then conducted several tests of the specificity of our severance payments and notice period measures. A small number of countries implemented changes to severance payment and notice period legislation during the periods under study, so we re‐estimated our models excluding countries that altered their legislation (Supporting Information 14). To account for an implementation delay in countries that introduced changes, we also re‐estimated our models using 1‐year lagged severance payment and notice period measures (Supporting Information 15). We also re‐estimated our models incorporating severance payments and notice periods together to test whether these associations were independent of one another (Supporting Information 16–18). Although our original models include unemployment spending, including spending on unemployment benefits, we also re‐estimated our results incorporating alternative labour market support programmes, including active labour market program spending (Supporting Information 19). In all cases, the results did not qualitatively change.

## 5 DISCUSSION

This paper has explored the relationship between employment status and health and how this association is structured by legal rules that re‐calibrate power asymmetries in setting dismissal conditions. Consistent with previous research (Paul & Moser, 2009), we found that employment status affects health. But, we also found that the link between employment status and health was modified by institutions that regulate dismissal conditions and associated power asymmetries. The broader economic context also appeared to be partially important, as this protective association was attenuated by the Great Recession. We also observed that dismissal regulations reduced the likelihood of a health decline and the risk of experiencing a rise in financial strain and that changes to financial strain were correlated with the likelihood of a health decline.

Four important insights can be drawn from our study. First, we found that both forms of dismissal legislation—severance payments and notice periods—had a protective association with health independent of welfare‐regime type, spending on unemployment, including cash benefits and spending on active labour market programmes, and the level of economic development (Paul & Moser, 2009). Most strikingly, in those countries where people were entitled to the largest severance payments, job loss had no statistically identifiable association with worsening health compared with those who remained in employment. These findings underscore the importance of considering and empirically investigating how the social determinants of labour market participants' health are structured by additional institutional arrangements, beyond welfare regimes, and how institutional theories of precariousness can provide insights into which additional institutions may be influential.

Second, institutional rules that redress power asymmetries in setting dismissal conditions appear to have a protective association with labour market participants' health (Hypothesis 1). Specifically, employed and recently unemployed workers were less likely to report a decline in self‐reported health where the legal minimum severance payments and notice periods for dismissal were larger (Hypothesis 2). Despite their different hypothesized mechanisms, these associations were substantively comparable. Third, these associations appear to be partially mediated by changes to financial strain as we observe that (a) dismissal legislation reduce the likelihood of a health decline and the risk of experiencing financial strain and that (a) financial strain is correlated with the likelihood of a health decline (Hypothesis 4). Taken together, these two findings suggest that severance payments and notice period legislation protect labour market participants' health by preventing a rise financial strain. Additional mechanisms may also play a role, such as facilitating adaptation to job loss by providing the resources and time for pursuing a career change. These findings may also extend to overall health inequalities, which are, in part, explained by labour market trajectories (Marmot, 2015; Olafsdottir, 2007).

Fourth, the institutions that regulate dismissal conditions and protect health may be sensitive to changes in the wider economic environment and associated changes to wages, insecurity, and public policy but only partially so (Hypothesis 3). We found that the Great Recession attenuated the protective associations of dismissal legislation with health, suggesting that reduced compliance, rising insecurity, and reductions in social protection spending partially eroded its protective effects. Intriguingly, the recession did not completely eliminate the protective associations of dismissal legislation with health. This is somewhat surprising because the Great Recession resulted in large increases in job insecurity for the employed and the unemployed (International Labour Organization, 2015). Of course, the nature and the depth of recessions experienced by countries varied in our sample and so more needs to be done to explore how this a recession's depth alters how the policies function. But, even after accounting for some of these differences—by measuring both the change in GDP and the change in unemployment—our results remain stable.

Our study has several limitations that we attempted to address. First, the OECD measures of dismissal legislation are imprecise, and we group countries into quartiles. The OECD's measure is based on legislation on the books, not necessarily those as implemented, which could create a source of measurement error. Second, to the extent possible, we corrected for sociodemographic differences in survey composition across waves, but there remains potential for unobserved heterogeneity. Third, although it is possible that there is a “healthy worker effect,” so that ill workers were more likely to lose jobs, our results were robust to the inclusion of a baseline SRH control, and our models adjusted for the impact of time‐invariant unobserved factors that could affect SRH. Fourth, results from mediation analysis in non‐experimental settings are often uncertain because such analyses require additional assumptions that are difficult to confirm (Gelman and Hill, 2007). Despite these challenges, our results are consistent with a theoretically plausible causal pathway.

Fifth, we do not have a direct measure of insecurity in these data. Despite this, we do find that dismissal legislation is associated with financial strain, one possible dimension and measure of insecurity. Sixth, there may be alternative sources of dismissal protection that we have not adjusted for due to data limitations, such as workplace unionization, and types of dismissal protection are likely to be positively interrelated. However, we controlled for both passive and active labour market supports, and, in models including both severance payments and notice periods, both factors were independently statistically significant. Importantly, Scandinavian countries belong to both high and low‐severance payment quartiles, suggesting that our results are not driven by welfare policies common in the Scandinavian context that are not captured by the index of dismissal legislation.

Seventh, our findings may apply to securely employed insiders only. Our results were consistent in alternative specifications excluding temporarily employed persons, suggesting that their inclusion in our analytic sample did not influence our results. It remains possible, however, that the legislation granted to outsiders is less generous in countries with stronger employment protection, representing a form of “dualization,” which differentiates the rights, entitlements, and services provided to different categories of recipients (Emmenegger et al., 2012). Whether or not, this applies here is an empirical question as “dualization without increasing divides is possible if outsider policies are generous” (Emmenegger and Hausermann 2012, p.12). Alternatively, the protections may not vary in generosity but still benefit insiders more than outsiders. This may happen because they do not provide an adequate buffer against the additional vulnerabilities and insecurities faced by outsiders, such as elevated stress and financial strain. Evaluating these questions is an important priority for future research.

Finally, dismissal legislation increases the costs of firing an employee, potentially increasing a firm's reluctance to take on new staff and making labour market entry more difficult for the long‐term unemployed. This has implications for research on “flexicurity” models, which combine social protection with flexible labour regulations. Severance and notice period legislation may enable firms to more easily dismiss workers, but this also appears to carry a wider social cost: It can undermine the health status of labour market participants, especially those who lose their jobs. Furthermore, recent evidence suggests that employment legislation (including severance payments and notice period legislation) does not necessarily affect employment rates or turnover (Blanchard & Portugal, 2001; Pissarides, 2001). In fact, these policies appear to increase the number of self‐employed workers and may thereby encourage entrepreneurship (Pissarides, 2001). In short, more generous protections appear to protect health without creating costly inflexibilities and adversely affecting those who are currently unemployed.

Hence, our findings have important policy implications. The IMF, OECD, and European Commission have recommended that governments restrict dismissal legislation (ILO, OECD, IMF, and World Bank, 2012). The findings from our study suggest that doing so may pose a health risk, suggesting the need for a cost–benefit analysis, which considers the impacts on health. Indeed, a number of recent studies have also challenged the potential macroeconomic benefits of reducing dismissal legislation (Betcherman, 2013; Deakin, Malmberg, & Sarkar, 2014; World Bank, 2013). Reducing dismissal legislation may therefore pose health risks whilst failing to yield the intended economic benefits.

## FUNDING INFORMATION

This work was funded by the European Commission Horizon 2020 Framework Programme, H2020 Excellent Science, H2020 European Research Council 313590, the John Fell Fund, University of Oxford, and the Wellcome Trust.

## Supporting information

Data S1. Supplemental Material 1: OECD employment protection legislation – conversion of raw scores to cardinal values
Supplemental Material 2: Countries in each severance payment and notice period quartile
Supplemental Material 3: Quartile coding description and sensitivity analysis quartile
Supplemental Material 4: Association between missing data and the probability of a health decline
Supplemental Material 5: Financial strain mediation analysis
Supplemental Material 6: Logistic regression estimation of association between severance payment and notice period quartiles and the probability of a health decline, separated by period
Supplemental Material 7: Effect of job loss and notice periods on probability of a health decline, estimated with job loss *x* notice period interaction
Supplemental Material 8: Effect of job loss and severance payments on predicted probability of a health decline, estimated with job loss *x* severance payment interaction
Supplemental Material 9: Associations between severance payment and notice period quartile and the probability of a health decline, before and during the recession, adjusting for welfare‐regime
Supplemental Material 10: Association between severance and notice period quartile and the probability of a health decline: linear probability models
Supplemental Material 11: Association between severance and notice period quartiles and the probability of a health decline, controlling for baseline self‐reported health
Supplemental Material 12: Association between severance and notice period quartiles and the probability of a health decline: including individuals that gained employment
Supplemental Material 13: Association between severance and notice period quartiles and the probability of a health decline: comparison of results excluding temporarily employed persons
Supplemental Material 14: Association between severance and notice period quartiles and the probability of a health decline: excluding countries that altered the level of employment protection
Supplemental Material 15: Association between severance and notice period quartiles and the probability of a health decline using lagged protection measures
Supplemental Material 16: Association between severance and notice period and the probability of a health decline: models incorporating both measures simultaneously
Supplemental Material 17: Predicted probability of a health decline, by severance payment quartile and incorporating notice period quartile control, before and during the recession
Supplemental Material 18: Predicted probability of a health decline, by notice and incorporating severance payment quartile control, before and during the recession
Supplemental Material 19: Association between severance and notice period quartiles and the probability of a health decline including control for active labour market programme spending (all years)

## Footnotes

*
*p* < 0.05;

**
*p* < 0.01;

***
*p* < 0.001. Standard errors in parentheses.
