# Supplementary material for: Employment relations and dismissal regulations: Does employment legislation protect the health of workers?
Source: Soc Policy Adm. 2019 Feb 14;53(7):939–57. doi: 10.1111/spol.12487 (PMC7983177; doi:10.1111/spol.12487)
Supplement: Supplementary file 1 — Data S1. Supplemental Material 1: OECD employment protection legislation – conversion of raw scores to cardinal values Supplemental Material 2: Countries in each severance payment and notice period quartile Supplemental Material 3: Quartile coding description and sensitivity analysis quartile Supplemental Material 4: Association between missing data and the probability of a health decline Supplemental Material 5: Financial strain mediation analysis Supplemental Material 6: Logistic regression estimation of association between severance payment and notice period quartiles and the probability of a health decline, separated by period Supplemental Material 7: Effect of job loss and notice periods on probability of a health decline, estimated with job loss x notice period interaction Supplemental Material 8: Effect of job loss and severance payments on predicted probability of a health decline, estimated with job loss x severance payment interaction Supplemental Material 9: Associations between severance payment and notice period quartile and the probability of a health decline, before and during the recession, adjusting for welfare‐regime Supplemental Material 10: Association between severance and notice period quartile and the probability of a health decline: linear probability models Supplemental Material 11: Association between severance and notice period quartiles and the probability of a health decline, controlling for baseline self‐reported health Supplemental Material 12: Association between severance and notice period quartiles and the probability of a health decline: including individuals that gained employment Supplemental Material 13: Association between severance and notice period quartiles and the probability of a health decline: comparison of results excluding temporarily employed persons Supplemental Material 14: Association between severance and notice period quartiles and the probability of a health decline: excluding countries that altered the level of [file SPOL-53-939-s001.docx]

**Supplemental Material**

Supplemental Material 1: OECD employment protection legislation – conversion of raw scores to cardinal values

Supplemental Material 2: Countries in each severance payment and notice period quartile

Supplemental Material 3: Quartile coding description and sensitivity analysis

quartile

Supplemental Material 4: Association between missing data and the probability of a health decline

Supplemental Material 5: Financial strain mediation analysis

Supplemental Material 6: Logistic regression estimation of association between severance payment and notice period quartiles and the probability of a health decline, separated by period

Supplemental Material 7: Effect of job loss and notice periods on probability of a health decline, estimated with job loss *x* notice period interaction

Supplemental Material 8: Effect of job loss and severance payments on predicted probability of a health decline, estimated with job loss *x* severance payment interaction

Supplemental Material 9: Associations between severance payment and notice period quartile and the probability of a health decline, before and during the recession, adjusting for welfare-regime

Supplemental Material 10: Association between severance and notice period quartile and the probability of a health decline: linear probability models

Supplemental Material 11: Association between severance and notice period quartiles and the probability of a health decline, controlling for baseline self-reported health

Supplemental Material 12: Association between severance and notice period quartiles and the probability of a health decline: including individuals that gained employment

Supplemental Material 13: Association between severance and notice period quartiles and the probability of a health decline: comparison of results excluding temporarily employed persons

Supplemental Material 14: Association between severance and notice period quartiles and the probability of a health decline: excluding countries that altered the level of employment protection

Supplemental Material 15: Association between severance and notice

period quartiles and the probability of a health decline using lagged protection measures

Supplemental Material 16: Association between severance and notice period and the probability of a health decline: models incorporating both measures simultaneously

Supplemental Material 17: Predicted probability of a health decline, by severance payment quartile and incorporating notice period quartile control, before and during the recession

Supplemental Material 18: Predicted probability of a health decline, by notice and incorporating severance payment quartile control, before and during the recession

Supplemental Material 19: Association between severance and notice period quartiles and the probability of a health decline including control for active labour market programme spending (all years)

Supplemental Material 1: OECD employment protection legislation – conversion of raw scores to cardinal values

|  |  | **Assignment of numerical strictness scores** | | | | | | |
| --- | --- | --- | --- | --- | --- | --- | --- | --- |
| **Length of notice period at** | **Original unit and short description** | **0** | **1** | **2** | **3** | **4** | **5** | **6** |
| 9 months tenure | Number of months | 0 | ≤0.4 | ≤0.8 | ≤1.2 | 1.6 | <2 | >2 |
| 4 years tenure | Number of months | 0 | ≤0.75 | ≤1.25 | <2 | <2.5 | <3.5 | ≥3.5 |
| 20 years tenure | Number of months | <1 | ≤2.75 | <5 | <7 | <9 | <11 | ≥11 |

|  |  | **Assignment of numerical strictness scores** | | | | | | |
| --- | --- | --- | --- | --- | --- | --- | --- | --- |
| **Severance pay at** | **Original unit and short description** | **0** | **1** | **2** | **3** | **4** | **5** | **6** |
| 9 months tenure | Months pay | 0 | ≤0.5 | ≤1 | ≤1.75 | ≤2.5 | <3 | ≥3 |
| 4 years tenure | Months pay | 0 | ≤0.5 | ≤1 | ≤2 | ≤3 | <4 | ≥4 |
| 20 years tenure | Months pay | 0 | ≤3 | ≤6 | ≤10 | ≤12 | ≤18 | >18 |

Supplemental Material 2: Countries in each Severance Payment and Notice Period Quartile

| **Severance Quartiles** | **Q1** | **Q2** | **Q3** | **Q4** |
| --- | --- | --- | --- | --- |
| *OECD Index* | *0* | *1-3* | *4-5* | *6-18* |
|  | Austria | Denmark | Belgium (2010) | Czech Republic  (2007-2010) |
|  | Belgium (2006-2009) | France | Czech Republic (2006) | Estonia  (2010) |
|  | Estonia (2005-2007) | Hungary | Estonia (2008-2009) | Netherlands |
|  | Finland | Ireland | Greece | Portugal |
|  | Iceland | Luxembourg  (2008-2010) | Poland | Spain |
|  | Italy | United Kingdom | Slovenia  (2008-2010) |  |
|  | Luxembourg  (2006-2007) |  | Slovakia |  |
|  | Norway |  |  |  |
|  | Sweden |  |  |  |
|  | Slovenia  (2006-2007) |  |  |  |
| *Total observations* | *113,017* | *82,981* | *79,541* | *72,461* |

| **Notice Quartiles** | **Q1** | **Q2** | **Q3** | **Q4** |
| --- | --- | --- | --- | --- |
| *OECD Index* | *0-6* | *7-8* | *9-10* | *11-17* |
|  | Austria | Estonia (2008-2009) | Greece | Belgium |
|  | Estonia  (2006-2007, 2010) | Finland | Italy | Czech Republic |
|  | Ireland | France | Poland | Denmark |
|  | Iceland  (2006-2007) | Hungary |  | Iceland  (2009-2010) |
|  | Luxembourg  (2006-2007) | Norway |  | Luxembourg (2009-2010) |
|  | Netherlands | Spain |  | Portugal |
|  | Slovenia |  |  | Sweden |
|  | United Kingdom |  |  | Slovakia |
| *Total observations* | *73,992* | *104,111* | *92,348* | *77,549* |

Supplemental Material 3: Quartile coding description and sensitivity analysis

Our decision to group countries into quartiles of dismissal legislation was based on three considerations. First, the raw dismissal legislation score has many values without any cases. For example, severance payment index scores ranged from 0 (indicated low severance) to 18 (indicating higher severance) but no countries had a score between 8 and 17. A coefficient based on estimating a 1-unit increase in the dismissal legislation score lacks validity if there are no observations within the data for approximately half the scale.

A second, related consideration is that some values of the index are occupied by a single country. For example, only Portugal has the severance payment index score, 18, only Netherlands has a score of 6, and only Denmark has a score of 2. Again, this complicates estimating a regression model using the continuous index score as our results could be driven by other factors that correlate with these individual country scores.

Finally, the raw OECD index is not intuitively interpretable as scores are assigned based on the generosity of minimum severance payments and notice periods for employees at three different tenure lengths.

These limitations suggest deploying the raw dismissal legislation score would potentially be misleading. In contrast, clustering countries by quartiles has a number of advantages:

1. It overcomes the sparse data problem, enabling us to adjust for covariates and better isolate the impact of dismissal legislation.

2. It retains the intuition of a scale by grouping together countries that may differ in other ways but which share similar severance payment/ notice period legislation scores.

3. It does not force us to make strong conclusions about the meaningfulness of small differences between countries.

4. Cutting the countries into quartiles maximizes the sample in each category but without using post-hoc rationalizations for why countries should be included in specific categories.

These advantages are generally recognized in the existing literature and is partly why comparative institutional analyses commonly cluster countries together in order to map institutional variations (Ebbinghaus 2012), such as Esping-Anderson’s welfare-regime typology (Esping‐Andersen 1990, p.37). Like welfare-regimes, grouping countries into dismissal protection quartiles facilitates a more straightforward interpretation whilst capturing variation in dismissal policies across a wide range of tenure lengths.

Of course, this raises the question as to whether our clustering procedure affected our results. To evaluate this we re-estimated our models using the raw score: the coefficients show a slightly weaker association with the dismissal legislation index as the scale of the unit change being modeled is 1 increase in the index score rather than a quartile increase, but the association is substantively comparable with our main findings.

*Table 2. Severance payment and notice period coefficients using raw severance payment and notice period scores*

| **Pre-recession** | **Model 1** | **Model 2** | **Model 3** | **Model 4** |
| --- | --- | --- | --- | --- |
| Severance payment index | 0.97 | 0.93 |  |  |
|  | (0.96 to 0.99) | (0.92 to 0.95) |  |  |
| Notice period index |  |  | 0.97 | 0.95 |
|  |  |  | (0.94 to 0.98) | (0.94 to 0.96) |
| Index coding | Raw score | Quartile | Raw score | Quartile |
| **Recession** | **Model 1** | **Model 2** | **Model 3** | **Model 4** |
| Severance payment index | 0.98 | 0.97 |  |  |
|  | (0.97 to 0.99) | (0.96 to 0.98) |  |  |
| Notice period index |  |  | 0.98 | 0.95 |
|  |  |  | (0.97 to 0.99) | (0.94 to 0.96) |
| Index coding | Raw score | Quartile | Raw score | Quartile |

*Notes:* 95% Confidence Intervals in parentheses. Exponentiated coefficients show association of a 1 unit increase in the variable in each column upon the odds of reporting a health decline.

Supplemental Material 4: Association between missing data and the probability of a health decline

| **Variable** | **Test of independence with probability of a health decline** | **p-value** |
| --- | --- | --- |
| Job loss | χ^2^(1) = 1.25 | 0.26 |
| Sex | χ^2^(1) = 1.32 | 0.25 |
| Education | χ^2^(1) = 22.49 | 0.00 |

*Notes:* test of independence with the probability of a health decline tests the hypothesis (H_1_) that reporting a health decline is not independent of having missing data on the explanatory variable, against the null hypothesis (H_0_) that having missing data on the explanatory variable is not independent reporting a health decline.

Supplemental Material 5: Financial strain mediation analysis

Our four-step test of the financial strain mechanism is based on a procedure proposed by Baron and Kenny (1986), as follows:

Step 1. Test for association between the probability of experiencing a decline in self-reported health and the severance payment or notice period quartile.

Step 2. Test for association between the severance payment or notice period quartile and financial strain.

Step 3. Test for association between the probability of experiencing a decline in self-reported health and financial strain.

Step 4. Test for association between the probability of experiencing a decline in self-reported health and the severance payment or notice period quartile, whilst adjusting for financial strain.

The goal of the first three steps is to establish whether relationships between the explanatory variable, outcome variable, and mediator actually exist. The logic of these steps is that it would be difficult to claim that the observed associations between severance payments/ notice periods and reporting a decline in self-reported health were mediated by financial strain if any of these relations were insignificant.

In step 4, the severance payment/ notice period variable and the financial strain variable are included simultaneously in a model predicting the probability of experiencing a decline in self-reported health. If the dismissal legislation is no longer significant when financial strain is controlled, the finding supports full mediation via financial strain; if the coefficient is attenuated, the finding supports partial mediation. If the financial strain variable is insignificant in this model, then mediation is not supported.

We have already established Step 1 in the results presented in the main text: there is an association between the dismissal legislation indices and reporting a health decline. Tables 1-3 below present the results from Steps 2-4 above. Tables 1-2 show that there is an association between the severance payment or notice period quartile and financial strain (Table 1, Step 2) and that there is an association between the probability of experiencing a decline in self-reported health and financial strain (Table 2, Step 3). Taken together, the results from Steps 1-3 (presented in the main text and below) show that relationships between the dismissal legislation indices, reporting a health decline, and financial strain all exist.

The results presented in Table 3 (Step 4) show that there is association between the probability of experiencing a decline in self-reported health and the severance payment or notice period quartile whilst adjusting for financial strain. As the dismissal legislation index coefficients are attenuated but not completely eroded in these models, these results suggest partial mediation via the financial strain mechanism.

*Table 1. Association between the severance payment or notice period quartile and financial strain.*

|  | **Odds of experiencing a health decline** |
| --- | --- |
| Fall into financial strain | 1.22*** |
|  | (0.03) |
| Male (dummy) | 1.02 |
|  | (0.02) |
| Age | 1.00 |
|  | (0.01) |
| Age Squared | 1.00 |
|  | (0.00) |
| *Education* |  |
| Lower Secondary | 1.05 |
|  | (0.4) |
| Upper Secondary | 0.97 |
|  | (0.03) |
| Post Secondary | 0.94* |
|  | (0.05) |
| Tertiary | 0.90** |
|  | (0.03) |
| Married | 0.94*** |
|  | (0.01) |
| *Occupation^b^* |  |
| Professionals | 0.93** |
|  | (0.02) |
| Technicians and assoc. professionals | 0.97 |
|  | (0.02) |
| Other assoc. professionals | 0.97 |
|  | (0.03) |
| Clerks | 1.00 |
|  | (0.03) |
| Service, shop and sales workers | 0.91* |
|  | (0.03) |
| Craft and trades workers | 0.99 |
|  | (0.03) |
| Plant and machine operators and assemblers | 1.03 |
|  | (0.03) |
| Elementary occupations | 1.07* |
|  | (0.03) |
| GDP | 1.004 |
|  | (0.00) |
| Change in GDP | 1.01 |
|  | (0.04) |
| Unemployment | 0.69 |
|  | (0.02) |
| Change in unemployment | 1.02* |
|  | (0.01) |
| Unemployment spending | 1.07*** |
|  | (0.01) |
| N | 113,090 |

*Notes:* P-values: * p<0.05 ; ** p<0.01; *** p<0.001. Standard errors in parentheses.

Exponentiated coefficients show association of 1 unit increase in the dependent variable on the odds of reporting a health decline. To facilitate interpretation we transform the GDP and unemployment variables as follows: coefficient of GDP shows association of 1000$ increase in GDP per capita; coefficient of unemployment, change in unemployment and change in GDP show association of a 10% increase; coefficient of unemployment spending shows association of 100 euro increase.

*Table 2. Association between the severance payment or notice period quartile and financial strain.*

|  | **Odds of it becoming difficult to make ends meet** | |
| --- | --- | --- |
|  | **Severance Payments** | **Notice Periods** |
| Male (dummy) | 1.00 | 1.00 |
|  | (0.02) | (0.02) |
| Age | 1.04*** | 1.04*** |
|  | (0.01) | (0.01) |
| Age Squared | 1.00*** | 1.00*** |
|  | (0.00) | (0.00) |
| *Education* |  |  |
| Lower Secondary | 0.97 | 0.98 |
|  | (0.05) | (0.05) |
| Upper Secondary | 1.09 | 1.10* |
|  | (0.05) | (0.05) |
| Post Secondary | 1.10 | 1.11 |
|  | (0.07) | (0.08) |
| Tertiary | 1.00 | 1.00 |
|  | (0.05) | (0.05) |
| Married | 0.94*** | 0.94*** |
|  | (0.01) | (0.01) |
| *Occupation^b^* |  |  |
| Professionals | 0.93** | 0.93* |
|  | (0.02) | (0.02) |
| Technicians and assoc. professionals | 0.97 | 0.9* |
|  | (0.02) | (0.02) |
| Other assoc. professionals | 0.97 | 0.97 |
|  | (0.03) | (0.03) |
| Clerks | 1.00 | 1.00 |
|  | (0.03) | (0.03) |
| Service, shop and sales workers | 0.91* | 0.91** |
|  | (0.03) | (0.03) |
| Craft and trades workers | 0.99 | 0.99 |
|  | (0.03) | (0.03) |
| Plant and machine operators and assemblers | 1.03 | 1.04 |
|  | (0.03) | (0.03) |
| Elementary occupations | 1.07* | 1.08** |
|  | (0.03) | (0.03) |
| GDP | 1.004 | 1.004 |
|  | (0.00) | (0.00) |
| Change in GDP | 1.01 | 1.01 |
|  | (0.04) | (0.04) |
| Unemployment | 0.69 | 0.69 |
|  | (0.02) | (0.02) |
| Change in unemployment | 1.02* | 1.02* |
|  | (0.01) | (0.01) |
| Unemployment spending | 1.07*** | 1.07*** |
|  | (0.01) | (0.01) |
| Job Loss | 1.66*** | 1.65*** |
|  | (0.10) | (0.10) |
| Recession | 0.96 | 0.95 |
|  | (0.04) | (0.05) |
| Severance Payment Quartile | 0.95** |  |
|  | (0.01) |  |
| Severance x Recession | 1.03 |  |
|  | (0.02) |  |
| Notice Payment Quartile |  | 0.91** |
|  |  | (0.01) |
| Notice x Recession |  | 1.05 |
|  |  | (0.03) |
| N | 113,090 | 113,090 |

*Notes:* P-values: * p<0.05 ; ** p<0.01; *** p<0.001

Standard errors in parentheses.

Exponentiated coefficients show association of 1 unit increase in the dependent variable on the odds of reporting a health decline. To facilitate interpretation we transform the GDP and unemployment variables as follows: coefficient of GDP shows association of 1000$ increase in GDP per capita; coefficient of unemployment, change in unemployment and change in GDP show association of a 10% increase; coefficient of unemployment spending shows association of 100 euro increase.

*Table 3. Association between the probability of a health decline and severance payment and notice period quartile, adjusting for financial strain*

|  | **Severance Payments** | **Notice Periods** |
| --- | --- | --- |
| Job Loss | 1.35*** | 1.30*** |
|  | (0.06) | (0.04) |
| Male (dummy) | 1.02 | 1.02 |
|  | (0.02) | (0.02) |
| Age | 1.01 | 1.01 |
|  | (0.006) | (0.006) |
| Age Squared | 0.99 | 1.00 |
|  | (0.00) | (0.00) |
| *Education* |  |  |
| Lower Secondary | 1.49 | 1.49 |
|  | (0.32) | (0.32) |
| Upper Secondary | 1.37* | 1.37* |
|  | (0.29) | (0.29) |
| Post Secondary | 1.32 | 1.32 |
|  | (0.29) | (0.29) |
| Tertiary | 1.27*** | 1.27*** |
|  | (0.27) | (0.27) |
| Married | 0.94*** | 0.94*** |
|  | (0.01) | (0.01) |
| *Occupation^b^* |  |  |
| Professionals | 0.93** | 0.93* |
|  | (0.02) | (0.02) |
| Technicians and assoc. professionals | 0.97 | 0.9* |
|  | (0.02) | (0.02) |
| Other assoc. professionals | 0.97 | 0.97 |
|  | (0.03) | (0.03) |
| Clerks | 1.00 | 1.00 |
|  | (0.03) | (0.03) |
| Service, shop and sales workers | 0.91* | 0.91** |
|  | (0.03) | (0.03) |
| Craft and trades workers | 0.99 | 0.99 |
|  | (0.03) | (0.03) |
| Plant and machine operators and assemblers | 1.03 | 1.04 |
|  | (0.03) | (0.03) |
| Elementary occupations | 1.07* | 1.08** |
|  | (0.03) | (0.03) |
| GDP | 1.003 | 1.004 |
|  | (0.001) | (0.001) |
| Change in GDP | 1.08 | 1.07 |
|  | (0.04) | (0.04) |
| Unemployment | 0.69 | 0.72 |
|  | (0.03) | (0.03) |
| Change in unemployment | 1.02*** | 1.01*** |
|  | (0.01) | (0.01) |
| Unemployment spending | 1.08*** | 1.07*** |
|  | (0.007) | (0.007) |
| Fall into financial strain | 1.22*** | 1.22*** |
|  | (0.03) | (0.03) |
| Severance Payment Quartile | 0.93***  (0.006) |  |
| Notice Period Quartile |  | 0.97*** |
|  |  | (0.006) |
| N | 175,326 | 172,674 |

*Notes:* P-values: * p<0.05 ; ** p<0.01; *** p<0.001. Standard errors in parentheses.

Exponentiated coefficients show association of 1 unit increase in the dependent variable on the odds of reporting a health decline. To facilitate interpretation we transform the GDP and unemployment variables as follows: coefficient of GDP shows association of 1000$ increase in GDP per capita; coefficient of unemployment, change in unemployment and change in GDP show association of a 10% increase; coefficient of unemployment spending shows association of 100 euro increase.

Supplemental Material 6: Logistic regression estimation of association between severance payment and notice period quartiles and the probability of a health decline, separated by period

|  | **Pre-Recession** | **Recession** | **Pre-Recession** | **Recession** |
| --- | --- | --- | --- | --- |
|  | **(2005-2007)** | **(2008-2010)** | **(2005-2007)** | **(2008-2010)** |
| Job Loss | 1.27*** | 1.30*** | 1.27*** | 1.30*** |
|  | (0.05) | (0.04) | (0.05) | (0.04) |
| Male (dummy) | 0.99 | 0.98 | 0.99 | 0.98 |
|  | (0.01) | (0.01) | (0.01) | (0.01) |
| Age | 1.01 | 1.01 | 1.01 | 1.01 |
|  | (0.004) | (0.004) | (0.004) | (0.004) |
| Age Squared | 1.00 | 1.00 | 1.00 | 1.00 |
|  | (0.00) | (0.00) | (0.00) | (0.00) |
| *Education^a^* |  |  |  |  |
| Lower Secondary | 1.05 | 1.07* | 1.07** | 1.07** |
|  | (0.03) | (0.03) | (0.03) | (0.03) |
| Upper Secondary | 1.00 | 1.01 | 1.02 | 1.02 |
|  | (0.02) | (0.02) | (0.02) | (0.02) |
| Post Secondary | 0.93* | 0.97 | 0.96 | 0.98 |
|  | (0.03) | (0.04) | (0.03) | (0.04) |
| Tertiary | 0.92 *** | 0.95* | 0.94** | 0.95* |
|  | (0.02) | (0.02) | (0.02) | (0.02) |
| Married | 0.94*** | 0.95*** | 0.94*** | 0.95*** |
|  | (0.01) | (0.01) | (0.01) | (0.01) |
| *Occupation^b^* |  |  |  |  |
| Professionals | 0.93** | 0.99 | 0.93* | 1.01 |
|  | (0.02) | (0.03) | (0.02) | (0.03) |
| Technicians and assoc. professionals | 0.97 | 1.04 | 0.9* | 1.05 |
|  | (0.02) | (0.03) | (0.02) | (0.03) |
| Other assoc. professionals | 0.97 | 1.02 | 0.97 | 1.02 |
|  | (0.03) | (0.03) | (0.03) | (0.03) |
| Clerks | 1.00 | 1.08** | 1.00 | 1.08** |
|  | (0.03) | (0.03) | (0.03) | (0.03) |
| Service, shop and sales workers | 0.91* | 0.97 | 0.91** | 0.98 |
|  | (0.03) | (0.04) | (0.03) | (0.04) |
| Craft and trades workers | 0.99 | 1.12*** | 0.99 | 1.12*** |
|  | (0.03) | (0.03) | (0.03) | (0.03) |
| Plant and machine operators and assemblers | 1.03 | 1.08* | 1.04 | 1.09*** |
|  | (0.03) | (0.03) | (0.03) | (0.03) |
| Elementary occupations | 1.07* | 1.16*** | 1.08** | 1.16*** |
|  | (0.03) | (0.04) | (0.03) | (0.04) |
| GDP | 1.001 | 1.01*** | 1.002*** | 1.01*** |
|  | (0.0007) | (0.0007) | (0.001) | (0.001) |
| Change in GDP | 1.06 | 1.03 | 0.98 | 1.02 |
|  | (0.03) | (0.02) | (0.03) | (0.02) |
| Unemployment | 1.01 | 0.84*** | 1.11** | 0.87*** |
|  | (0.03) | (0.02) | (0.04) | (0.03) |
| Change in unemployment | 1.07*** | 1.02*** | 1.04*** | 1.02*** |
|  | (0.01) | (0.004) | (0.01) | (0.004) |
| Unemployment spending | 1.11*** | 1.05*** | 1.07*** | 1.03*** |
|  | (0.01) | (0.005) | (0.005) | (0.005) |
| Severance Payment Quartile | 0.93*** | 0.97*** |  |  |
|  | (0.008) | (0.006) |  |  |
| Notice Period Quartile |  |  | 0.95*** | 0.95*** |
|  |  |  | (0.007) | (0.006) |
| N | 175,326 | 172,674 | 175,326 | 172,674 |

*Notes:* a: Education reference group is primary education. b: Occupation reference group is armed forces, legislators, senior officials and managers. P-values: * p<0.05 ; ** p<0.01; *** p<0.001. Standard errors in parentheses. Exponentiated coefficients show association of 1 unit increase in the dependent variable on the odds of reporting a health decline. To facilitate interpretation we transform the GDP and unemployment variables as follows: coefficient of GDP shows association of 1000$ increase in GDP per capita; coefficient of unemployment, change in unemployment and change in GDP show association of a 10% increase; coefficient of unemployment spending shows association of 100 euro increase.

Supplemental Material 7: Effect of job loss and notice periods on probability of a health decline, estimated with job loss *x* notice period interaction

| **Period** | **Short notice period** | **Long notice period** | **Difference** | **p-value** |
| --- | --- | --- | --- | --- |
| *No Recession* |  |  |  |  |
| Effect of job loss on probability of a health decline | 0.255  [0.232 – 0.278] | 0.201  [0.178 – 0.223] | 0.054  [0.016 – 0.092] | 0.005 |
| *Recession* |  |  |  |  |
| Effect of job loss on probability of a health decline | 0.223  [0.204 – 0.242] | 0.210  [0.192 – 0.229] | 0.013  [-0.019 – 0.044] | 0.437 |

*Notes:* Point estimates show predicted probability of a health decline with 95% confidence intervals in parentheses, estimated after estimating a logistic regression model with job loss *x* notice period quartile interaction. ‘Short notice period’ refers to the shortest notice period quartile; ‘long notice period’ refers to the highest quartile.

Supplemental Material 8: Effect of job loss and severance payments on predicted probability of a health decline, estimated with job loss *x* severance payment interaction

| **Period** | **Low level of severance payment** | **High level of severance payment** | **Difference** | **p-value** |
| --- | --- | --- | --- | --- |
| *No Recession* |  |  |  |  |
| Effect of job loss on probability of a health decline | 0.265  [0.245 – 0.286] | 0.185 [0.166 – 0.204] | 0.08  [0.049 – 0.111] | 0.000 |
| *Recession* |  | |  |  |
| Effect of job loss on probability of a health decline | 0.238  [0.219 – 0.257] | 0.198  [0.183 – 0.213] | 0.040  [0.013 – 0.067] | 0.004 |

*Notes*: Point estimates show predicted probability of a health decline with 95% confidence intervals in parentheses, computed after estimating a logistic regression model with job loss *x* severance payment quartile interaction. ‘Low level of severance payment’ refers to lowest severance payment quartile; ‘high level’ refers to the highest quartile.

Supplemental Material 9: Associations between severance payment and notice period quartile and the probability of a health decline, before and during the recession, adjusting for welfare-regime

|  | **Pre-Recession** | **Recession** | **Pre-Recession** | **Recession** |
| --- | --- | --- | --- | --- |
|  | **(2005-2007)** | **(2008-2010)** | **(2005-2007)** | **(2008-2010)** |
| Job Loss | 1.32*** | 1.33*** | 1.32*** | 1.33*** |
|  | (0.05) | (0.05) | (0.05) | (0.05) |
| Male (dummy) | 1.00 | 0.98 | 0.99 | 0.98 |
|  | (0.01) | (0.01) | (0.01) | (0.01) |
| Age | 1.00 | 1.01* | 1.00 | 1.01* |
|  | (0.004) | (0.004) | (0.004) | (0.004) |
| Age Squared | 1.00 | 1.00 | 1.00 | 1.00 |
|  | (0.00) | (0.00) | (0.00) | (0.00) |
| *Education* |  |  |  |  |
| Lower Secondary | 1.06 | 0.99 | 1.10*** | 1.02 |
|  | (0.03) | (0.03) | (0.03) | (0.03) |
| Upper Secondary | 0.95* | 0.87*** | 0.99 | 0.90*** |
|  | (0.02) | (0.02) | (0.02) | (0.02) |
| Post Secondary | 0.92* | 0.86** | 0.98 | 0.90* |
|  | (0.03) | (0.04) | (0.03) | (0.04) |
| Tertiary | 0.89*** | 0.82*** | 0.92** | 0.84* |
|  | (0.02) | (0.02) | (0.02) | (0.02) |
| Married | 0.94*** | 0.95*** | 0.94*** | 0.95*** |
|  | (0.01) | (0.01) | (0.01) | (0.01) |
| *Occupation^b^* |  |  |  |  |
| Professionals | 0.93** | 0.99 | 0.93* | 1.01 |
|  | (0.02) | (0.03) | (0.02) | (0.03) |
| Technicians and assoc. professionals | 0.97 | 1.04 | 0.9* | 1.05 |
|  | (0.02) | (0.03) | (0.02) | (0.03) |
| Other assoc. professionals | 0.97 | 1.02 | 0.97 | 1.02 |
|  | (0.03) | (0.03) | (0.03) | (0.03) |
| Clerks | 1.00 | 1.08** | 1.00 | 1.08** |
|  | (0.03) | (0.03) | (0.03) | (0.03) |
| Service, shop and sales workers | 0.91* | 0.97 | 0.91** | 0.98 |
|  | (0.03) | (0.04) | (0.03) | (0.04) |
| Craft and trades workers | 0.99 | 1.12*** | 0.99 | 1.12*** |
|  | (0.03) | (0.03) | (0.03) | (0.03) |
| Plant and machine operators and assemblers | 1.03 | 1.08* | 1.04 | 1.09*** |
|  | (0.03) | (0.03) | (0.03) | (0.03) |
| Elementary occupations | 1.07* | 1.16*** | 1.08** | 1.16*** |
|  | (0.03) | (0.04) | (0.03) | (0.04) |
| GDP | 1.06 | 1.03 | 0.98 | 1.02 |
|  | (0.03) | (0.02) | (0.03) | (0.02) |
| Change in GDP | 1.01 | 0.84*** | 1.11** | 0.87*** |
|  | (0.03) | (0.02) | (0.04) | (0.03) |
| Unemployment | 1.07*** | 1.02*** | 1.04*** | 1.02*** |
|  | (0.01) | (0.004) | (0.01) | (0.004) |
| Change in unemployment | 1.11*** | 1.05*** | 1.07*** | 1.03*** |
|  | (0.01) | (0.005) | (0.005) | (0.005) |
| Unemployment spending | 1.06 | 1.03 | 0.98 | 1.02 |
|  | (0.03) | (0.02) | (0.03) | (0.02) |
| *Welfare regime^a^* |  |  |  |  |
| Bismarkian | 1.01 | 1.10*** | 0.90*** | 0.99 |
|  | (0.02) | (0.03) | (0.02) | (0.02) |
| Anglo-Saxon | 1.05 | 1.00 | 0.90** | 0.90** |
|  | (0.03) | (0.04) | (0.03) | (0.03) |
| Southern Europe | 0.82*** | 0.64*** | 0.84*** | 0.60*** |
|  | (0.02) | (0.02) | (0.03) | (0.02) |
| Eastern Europe | 1.28*** | 0.92** | 1.24*** | 0.83*** |
|  | (0.04) | (0.03) | (0.04) | (0.02) |
| Severance Payment Quartile | 0.92*** | 0.94*** |  |  |
|  | (0.007) | (0.001) |  |  |
| Notice Period Quartile |  |  | 0.96*** | 0.98** |
|  |  |  | (0.008) | (0.006) |
| N | 175,326 | 172,674 | 175,326 | 172,674 |

*Notes:* a: welfare-regime coding follows Bambra and Eikemo’s (2009) classification. Scandinavian is the reference category. P-values: * p<0.05 ; ** p<0.01; *** p<0.001. Standard errors in parentheses. Exponentiated coefficients show association of 1 unit increase in the dependent variable on the odds of reporting a health decline. To facilitate interpretation we transform the GDP and unemployment variables as follows: coefficient of GDP shows association of 1000$ increase in GDP per capita; coefficient of unemployment, change in unemployment and change in GDP show association of a 10% increase; coefficient of unemployment spending shows association of 100 euro increase.

Supplemental Material 10: Association between severance and notice period quartile and the probability of a health decline: OLS results

|  | **Severance Payments** | **Notice Periods** |
| --- | --- | --- |
| Job Loss | 0.03*** | 0.03*** |
|  | (0.002) | (0.002) |
| Male (dummy) | -0.002 | -0.002 |
|  | (0.001) | (0.001) |
| Age | 0.001** | 0.001** |
|  | (0.00) | (0.00) |
| Age Squared | -0.00 | -0.00 |
|  | (0.00) | (0.00) |
| *Education* |  |  |
| Lower Secondary | 0.04** | 0.01* |
|  | (0.003) | (0.003) |
| Upper Secondary | -0.02* | -0.001* |
|  | (0.002) | (0.002) |
| Post Secondary | -0.01** | -0.01* |
|  | (0.004) | (0.004) |
| Tertiary | -0.01*** | -0.01*** |
|  | (0.003) | (0.003) |
| Married | 0.94*** | 0.94*** |
|  | (0.01) | (0.01) |
| *Occupation^b^* |  |  |
| Professionals | 0.93** | 0.93* |
|  | (0.02) | (0.02) |
| Technicians and assoc. professionals | 0.97 | 0.9* |
|  | (0.02) | (0.02) |
| Other assoc. professionals | 0.97 | 0.97 |
|  | (0.03) | (0.03) |
| Clerks | 1.00 | 1.00 |
|  | (0.03) | (0.03) |
| Service, shop and sales workers | 0.91* | 0.91** |
|  | (0.03) | (0.03) |
| Craft and trades workers | 0.99 | 0.99 |
|  | (0.03) | (0.03) |
| Plant and machine operators and assemblers | 1.03 | 1.04 |
|  | (0.03) | (0.03) |
| Elementary occupations | 1.07* | 1.08** |
|  | (0.03) | (0.03) |
| GDP | 0.004*** | 0.004*** |
|  | (0.00008) | (0.00008) |
| Change in GDP | 0.01*** | 0.01*** |
|  | (0.003) | (0.003) |
| Unemployment | -0.04*** | -0.04*** |
|  | (0.0002) | (0.0002) |
| Change in unemployment | 0.002** | 0.002** |
|  | (0.0005) | (0.0005) |
| Unemployment spending | 0.01*** | 0.01*** |
|  | (0.0005) | (0.0005) |
| Severance Payment Quartile | -0.006*** |  |
|  | (0.001) |  |
| Notice Period Quartile |  | -0.005*** |
|  |  | (0.001) |
| Constant | 0.17*** | 0.16*** |
|  | (0.01) | (0.01) |
| N | 348,000 | 348,000 |

*Notes:* P-values: * p<0.05 ; ** p<0.01; *** p<0.001. Standard errors in parentheses. Exponentiated coefficients show association of 1 unit increase in the dependent variable on the odds of reporting a health decline. To facilitate interpretation we transform the GDP and unemployment variables as follows: coefficient of GDP shows association of 1000$ increase in GDP per capita; coefficient of unemployment, change in unemployment and change in GDP show association of a 10% increase; coefficient of unemployment spending shows association of 100 euro increase.

Supplemental Material 11: Association between severance and notice period quartiles and the probability of a health decline: incorporating baseline self-reported health

|  | **Severance Payments** | **Notice Periods** |
| --- | --- | --- |
| Job Loss | 1.64*** | 1.62*** |
|  | (0.08) | (0.08) |
| Male (dummy) | 0.94*** | 0.94*** |
|  | (0.02) | (0.02) |
| Age | 1.04*** | 1.05*** |
|  | (0.01) | (0.01) |
| Age Squared | 1.00*** | 1.00*** |
|  | (0.00) | (0.00) |
| *Education* |  |  |
| Lower Secondary | 0.92* | 0.93* |
|  | (0.03) | (0.03) |
| Upper Secondary | 0.73*** | 0.74*** |
|  | (0.02) | (0.02) |
| Post Secondary | 0.66*** | 0.67*** |
|  | (0.03) | (0.03) |
| Tertiary | 0.55** | 0.56*** |
|  | (0.02) | (0.02) |
| Married | 0.94*** | 0.94*** |
|  | (0.01) | (0.01) |
| *Occupation^b^* |  |  |
| Professionals | 0.93** | 0.93* |
|  | (0.02) | (0.02) |
| Technicians and assoc. professionals | 0.97 | 0.9* |
|  | (0.02) | (0.02) |
| Other assoc. professionals | 0.97 | 0.97 |
|  | (0.03) | (0.03) |
| Clerks | 1.00 | 1.00 |
|  | (0.03) | (0.03) |
| Service, shop and sales workers | 0.91* | 0.91** |
|  | (0.03) | (0.03) |
| Craft and trades workers | 0.99 | 0.99 |
|  | (0.03) | (0.03) |
| Plant and machine operators and assemblers | 1.03 | 1.04 |
|  | (0.03) | (0.03) |
| Elementary occupations | 1.07* | 1.08** |
|  | (0.03) | (0.03) |
| GDP | 0.99*** | 0.99*** |
|  | (0.001) | (0.001) |
| Change in GDP | 1.54*** | 1.54*** |
|  | (0.07) | (0.07) |
| Unemployment | 0.66*** | 0.66*** |
|  | (0.02) | (0.02) |
| Change in unemployment | 1.01 | 1.01 |
|  | (0.008) | (0.008) |
| Unemployment spending | 1.12** | 1.12** |
|  | (0.007) | (0.007) |
| Lagged health (2009) | 0.26*** | 0.26*** |
|  | (0.004) | (0.004) |
| Severance Payment Quartile | 0.98* |  |
|  | (0.01) |  |
| Notice Period Quartile |  | 0.96*** |
|  |  | (0.01) |
| N | 118,402 | 118,402 |

*Notes:* P-values: * p<0.05 ; ** p<0.01; *** p<0.001. Standard errors in parentheses. Exponentiated coefficients show association of 1 unit increase in the dependent variable on the odds of reporting a health decline. To facilitate interpretation we transform the GDP and unemployment variables as follows: coefficient of GDP shows association of 1000$ increase in GDP per capita; coefficient of unemployment, change in unemployment and change in GDP show association of a 10% increase; coefficient of unemployment spending shows association of 100 euro increase.

Supplemental Material 12: Predicted probability of a health decline, by severance payment quartile and incorporating those who gain employment between survey waves coded with employed persons

|  | **Severance Payments** | **Notice Periods** |
| --- | --- | --- |
| Job Loss | 1.29*** | 1.29*** |
|  | (0.03) | (0.03) |
| Male (dummy) | 0.99 | 0.99 |
|  | (0.01) | (0.01) |
| Age | 1.01* | 1.01* |
|  | (0.003) | (0.003) |
| Age Squared | 1.00 | 1.00 |
|  | (0.00) | (0.00) |
| Married (dummy) | 0.94*** | 0.94*** |
|  | (0.01) | (0.01) |
| *Education* |  |  |
| Lower Secondary | 1.06 | 1.06 |
|  | (0.03) | (0.03) |
| Upper Secondary | 0.95* | 0.95* |
|  | (0.02) | (0.02) |
| Post Secondary | 0.92* | 0.92* |
|  | (0.03) | (0.03) |
| Tertiary | 0.89*** | 0.89*** |
|  | (0.02) | (0.02) |
| Married | 0.94*** | 0.94*** |
|  | (0.01) | (0.01) |
| *Occupation^b^* |  |  |
| Professionals | 0.93** | 0.93* |
|  | (0.02) | (0.02) |
| Technicians and assoc. professionals | 0.97 | 0.9* |
|  | (0.02) | (0.02) |
| Other assoc. professionals | 0.97 | 0.97 |
|  | (0.03) | (0.03) |
| Clerks | 1.00 | 1.00 |
|  | (0.03) | (0.03) |
| Service, shop and sales workers | 0.91* | 0.91** |
|  | (0.03) | (0.03) |
| Craft and trades workers | 0.99 | 0.99 |
|  | (0.03) | (0.03) |
| Plant and machine operators and assemblers | 1.03 | 1.04 |
|  | (0.03) | (0.03) |
| Elementary occupations | 1.07* | 1.08** |
|  | (0.03) | (0.03) |
| GDP | 1.06 | 1.06 |
|  | (0.03) | (0.03) |
| Change in GDP | 1.01 | 1.01 |
|  | (0.03) | (0.03) |
| Unemployment | 1.07*** | 1.07*** |
|  | (0.01) | (0.01) |
| Change in unemployment | 1.11*** | 1.11*** |
|  | (0.01) | (0.01) |
| Unemployment spending | 1.06 | 1.06 |
|  | (0.03) | (0.03) |
| Severance Payment Quartile | 0.96*** |  |
|  | (0.004) |  |
| Notice Period Quartile |  | 0.97*** |
|  |  | (0.004) |
| N | 348,000 | 348,000 |

*Notes:* P-values: * p<0.05 ; ** p<0.01; *** p<0.001. Standard errors in parentheses. Exponentiated coefficients show association of 1 unit increase in the dependent variable on the odds of reporting a health decline. To facilitate interpretation we transform the GDP and unemployment variables as follows: coefficient of GDP shows association of 1000$ increase in GDP per capita; coefficient of unemployment, change in unemployment and change in GDP show association of a 10% increase; coefficient of unemployment spending shows association of 100 euro increase.

Supplemental Material 13: Association between severance and notice period quartiles and the probability of a health decline: comparison of results excluding temporarily employed persons

| **Pre-recession** | **Model 1** | **Model 2** | **Model 3** | **Model 4** |
| --- | --- | --- | --- | --- |
| Severance payment quartile | 0.93 | 0.95 |  |  |
|  | (0.91 to 0.93) | (0.94 to 0.97) |  |  |
| Notice period quartile |  |  | 0.95 | 0.94 |
|  |  |  | (0.94 to 0.97) | (0.93 to 0.96) |
| Includes temporarily employed persons? | Y | N | Y | N |
| **Recession** | **Model 1** | **Model 2** | **Model 3** | **Model 4** |
| Severance payment quartile | 0.96 | 0.95 |  |  |
|  | (0.95 to 0.98) | (0.94 to 0.98) |  |  |
| Notice period quartile |  |  | 0.96 | 0.96 |
|  |  |  | (0.95 to 0.97) | (0.94 to 0.96) |
| Marital status & employment sector controls? | Y | N | Y | N |

*Notes*: Confidence intervals in in parentheses. Exponentiated coefficients show association of 1 unit increase in the dependent variable on the odds of reporting a health decline.

Supplemental Material 14: Association between severance and notice period quartiles and the probability of a health decline: excluding countries that altered the level of employment protection

|  | **Severance Payments** | **Notice Periods** |
| --- | --- | --- |
| Job Loss | 1.35*** | 1.35*** |
|  | (0.06) | (0.06) |
| Male (dummy) | 1.01 | 1.01 |
|  | (0.02) | (0.02) |
| Age | 1.01 | 1.01 |
|  | (0.006) | (0.006) |
| Age Squared | 1.00 | 1.00 |
|  | (0.00) | (0.00) |
| Married (dummy) | 0.95** | 0.95** |
|  | (0.02) | (0.02) |
| *Education* |  |  |
| Lower Secondary | 1.06 | 1.06 |
|  | (0.03) | (0.03) |
| Upper Secondary | 0.95* | 0.95* |
|  | (0.02) | (0.02) |
| Post Secondary | 0.92* | 0.92* |
|  | (0.03) | (0.03) |
| Tertiary | 0.89*** | 0.89*** |
|  | (0.02) | (0.02) |
| Married | 0.94*** | 0.94*** |
|  | (0.01) | (0.01) |
| *Occupation^b^* |  |  |
| Professionals | 0.93** | 0.93* |
|  | (0.02) | (0.02) |
| Technicians and assoc. professionals | 0.97 | 0.9* |
|  | (0.02) | (0.02) |
| Other assoc. professionals | 0.97 | 0.97 |
|  | (0.03) | (0.03) |
| Clerks | 1.00 | 1.00 |
|  | (0.03) | (0.03) |
| Service, shop and sales workers | 0.91* | 0.91** |
|  | (0.03) | (0.03) |
| Craft and trades workers | 0.99 | 0.99 |
|  | (0.03) | (0.03) |
| Plant and machine operators and assemblers | 1.03 | 1.04 |
|  | (0.03) | (0.03) |
| Elementary occupations | 1.07* | 1.08** |
|  | (0.03) | (0.03) |
| GDP | 1.06 | 1.06 |
|  | (0.03) | (0.03) |
| Change in GDP | 1.01 | 1.01 |
|  | (0.03) | (0.03) |
| Unemployment | 1.07*** | 1.07*** |
|  | (0.01) | (0.01) |
| Change in unemployment | 1.11*** | 1.11*** |
|  | (0.01) | (0.01) |
| Unemployment spending | 1.06 | 1.06 |
|  | (0.03) | (0.03) |
| Severance Payment Quartile | 0.98* |  |
|  | (0.01) |  |
| Notice Period Quartile |  | 0.96*** |
|  |  | (0.01) |
| N | 112,168 | 112,168 |

*Notes*: P-values: * p<0.05 ; ** p<0.01; *** p<0.001. Standard errors in parentheses. Exponentiated coefficients show association of 1 unit increase in the dependent variable on the odds of reporting a health decline. To facilitate interpretation we transform the GDP and unemployment variables as follows: coefficient of GDP shows association of 1000$ increase in GDP per capita; coefficient of unemployment, change in unemployment and change in GDP show association of a 10% increase; coefficient of unemployment spending shows association of 100 euro increase.

Supplemental Material 15: Association between severance and notice period quartiles and the probability of a health decline: lagged measures

|  | **Severance Payments** | **Notice Periods** |
| --- | --- | --- |
| Job Loss | 1.35*** | 1.35*** |
|  | (0.06) | (0.06) |
| Male (dummy) | 1.02 | 1.02 |
|  | (0.02) | (0.02) |
| Age | 1.01 | 1.01 |
|  | (0.006) | (0.006) |
| Age Squared | 1.00 | 1.00 |
|  | (0.00) | (0.00) |
| *Education* |  |  |
| Lower Secondary | 1.04 | 1.03 |
|  | (0.03) | (0.03) |
| Upper Secondary | 0.95 | 0.95 |
|  | (0.03) | (0.03) |
| Post Secondary | 0.91 | 0.91 |
|  | (0.04) | (0.04) |
| Tertiary | 0.88*** | 0.88*** |
|  | (0.03) | (0037) |
| Married | 0.94*** | 0.94*** |
|  | (0.01) | (0.01) |
| *Occupation^b^* |  |  |
| Professionals | 0.93** | 0.93* |
|  | (0.02) | (0.02) |
| Technicians and assoc. professionals | 0.97 | 0.9* |
|  | (0.02) | (0.02) |
| Other assoc. professionals | 0.97 | 0.97 |
|  | (0.03) | (0.03) |
| Clerks | 1.00 | 1.00 |
|  | (0.03) | (0.03) |
| Service, shop and sales workers | 0.91* | 0.91** |
|  | (0.03) | (0.03) |
| Craft and trades workers | 0.99 | 0.99 |
|  | (0.03) | (0.03) |
| Plant and machine operators and assemblers | 1.03 | 1.04 |
|  | (0.03) | (0.03) |
| Elementary occupations | 1.07* | 1.08** |
|  | (0.03) | (0.03) |
| GDP | 1.06 | 1.06 |
|  | (0.03) | (0.03) |
| Change in GDP | 1.01 | 1.01 |
|  | (0.03) | (0.03) |
| Unemployment | 1.07*** | 1.07*** |
|  | (0.01) | (0.01) |
| Change in unemployment | 1.11*** | 1.11*** |
|  | (0.01) | (0.01) |
| Unemployment spending | 1.06 | 1.06 |
|  | (0.03) | (0.03) |
| Severance Payment Quartile | 0.97* |  |
|  | (0.01) |  |
| Notice Period Quartile |  | 0.96*** |
|  |  | (0.01) |
| N | 118,402 | 118,402 |

*Notes*: Model uses lagged measures of severance payment and notice period quartiles. P-values: * p<0.05 ; ** p<0.01; *** p<0.001. Standard errors in parentheses. Exponentiated coefficients show association of 1 unit increase in the dependent variable on the odds of reporting a health decline. To facilitate interpretation we transform the GDP and unemployment variables as follows: coefficient of GDP shows association of 1000$ increase in GDP per capita; coefficient of unemployment, change in unemployment and change in GDP show association of a 10% increase; coefficient of unemployment spending shows association of 100 euro increase.

Supplemental Material 16: Association between severance and notice period and the probability of a health decline: models incorporating both measures simultaneously

|  | **Pre-Recession** | **Recession** |
| --- | --- | --- |
| Job Loss | 1.27*** | 1.30*** |
|  | (0.05) | (0.04) |
| Male (dummy) | 0.99 | 0.98 |
|  | (0.01) | (0.01) |
| Age | 1.01 | 1.01 |
|  | (0.004) | (0.004) |
| Age Squared | 0.99 | 0.99 |
|  | (0.00) | (0.00) |
| *Education* |  |  |
| Lower Secondary | 1.04 | 1.06* |
|  | (0.03) | (0.03) |
| Upper Secondary | 1.00 | 1.00 |
|  | (0.02) | (0.02) |
| Post Secondary | 0.92* | 0.96 |
|  | (0.03) | (0.04) |
| Tertiary | 0.92*** | 0.94* |
|  | (0.02) | (0.02) |
| Married | 0.94*** | 0.94*** |
|  | (0.01) | (0.01) |
| *Occupation^b^* |  |  |
| Professionals | 0.93** | 0.93* |
|  | (0.02) | (0.02) |
| Technicians and assoc. professionals | 0.97 | 0.9* |
|  | (0.02) | (0.02) |
| Other assoc. professionals | 0.97 | 0.97 |
|  | (0.03) | (0.03) |
| Clerks | 1.00 | 1.00 |
|  | (0.03) | (0.03) |
| Service, shop and sales workers | 0.91* | 0.91** |
|  | (0.03) | (0.03) |
| Craft and trades workers | 0.99 | 0.99 |
|  | (0.03) | (0.03) |
| Plant and machine operators and assemblers | 1.03 | 1.04 |
|  | (0.03) | (0.03) |
| Elementary occupations | 1.07* | 1.08** |
|  | (0.03) | (0.03) |
| GDP | 1.06 | 1.06 |
|  | (0.03) | (0.03) |
| Change in GDP | 1.01 | 1.01 |
|  | (0.03) | (0.03) |
| Unemployment | 1.07*** | 1.07*** |
|  | (0.01) | (0.01) |
| Change in unemployment | 1.11*** | 1.11*** |
|  | (0.01) | (0.01) |
| Unemployment spending | 1.06 | 1.06 |
|  | (0.03) | (0.03) |
| Severance Payment Quartile | 0.94*** | 0.97*** |
|  | (0.00) | (0.01) |
| Notice Period Quartile | 0.96*** | 0.95*** |
|  | (0.01) | (0.01) |
| *N* | 175,326 | 172,674 |

*Notes* P-values: * p<0.05 ; ** p<0.01; *** p<0.001. Standard errors in parentheses. Exponentiated coefficients show association of 1 unit increase in the dependent variable on the odds of reporting a health decline To facilitate interpretation we transform the GDP and unemployment variables as follows: coefficient of GDP shows association of 1000$ increase in GDP per capita; coefficient of unemployment, change in unemployment and change in GDP show association of a 10% increase; coefficient of unemployment spending shows association of 100 euro increase.

Supplemental Material 17: Predicted probability of a health decline, by severance payment quartile and incorporating notice period quartile control, before and during the recession

*Before the recession*

**

*Notes:* Examples of countries in each quartile: Q1 – Italy, Norway. Q2 – Denmark, Hungary. Q3 – Greece, Slovakia. Q4 – Netherlands, Spain. Predicted probabilities computed after estimating full model with severance payment *x* job loss interaction.

*During the recession*

**

*Notes:* Examples of countries in each quartile: Q1 – Italy, Norway. Q2 – Denmark, Hungary. Q3 – Greece, Slovakia. Q4 – Netherlands, Spain. Predicted probabilities computed after estimating full model with severance payment *x* job loss interaction.

Supplemental Material 18: Predicted probability of a health decline, by notice period quartile and incorporating severance payment quartile control, before and during the recession

*Before the recession*

*Notes:* Examples of countries in each quartile: Q1 – Italy, Norway. Q2 – Denmark, Hungary. Q3 – Greece, Slovakia. Q4 – Netherlands, Spain. Predicted probabilities computed after estimating full model with severance payment *x* job loss interaction.

*During the recession*

**

*Notes:* Examples of countries in each quartile: Q1 – Italy, Norway. Q2 – Denmark, Hungary. Q3 – Greece, Slovakia. Q4 – Netherlands, Spain. Predicted probabilities computed after estimating full model with severance payment *x* job loss interaction.

Supplemental Material 19: Association between severance and notice period quartiles and the probability of a health decline including control for active labour market programme spending (all years) ^a^

|  | **Severance Payments** | **Notice Periods** |
| --- | --- | --- |
| Job Loss | 1.32*** | 1.32*** |
|  | (0.05) | (0.05) |
| Male (dummy) | 0.99 | 0.99 |
|  | (0.01) | (0.01) |
| Age | 1.01 | 1.01 |
|  | (0.004) | (0.004) |
| Age Squared | 0.99 | 0.99 |
|  | (0.00) | (0.00) |
| *Education* |  |  |
| Lower Secondary | 1.06* | 1.08*** |
|  | (0.03) | (0.02) |
| Upper Secondary | 0.99 | 0.99 |
|  | (0.02) | (0.02) |
| Post Secondary | 0.93* | 0.95 |
|  | (0.04) | (0.03) |
| Tertiary | 0.92*** | 0.93*** |
|  | (0.02) | (0.02) |
| Married | 0.94*** | 0.94*** |
|  | (0.01) | (0.01) |
| *Occupation^b^* |  |  |
| Professionals | 0.93** | 0.93* |
|  | (0.02) | (0.02) |
| Technicians and assoc. professionals | 0.97 | 0.9* |
|  | (0.02) | (0.02) |
| Other assoc. professionals | 0.97 | 0.97 |
|  | (0.03) | (0.03) |
| Clerks | 1.00 | 1.00 |
|  | (0.03) | (0.03) |
| Service, shop and sales workers | 0.91* | 0.91** |
|  | (0.03) | (0.03) |
| Craft and trades workers | 0.99 | 0.99 |
|  | (0.03) | (0.03) |
| Plant and machine operators and assemblers | 1.03 | 1.04 |
|  | (0.03) | (0.03) |
| Elementary occupations | 1.07* | 1.08** |
|  | (0.03) | (0.03) |
| GDP | 1.06 | 1.06 |
|  | (0.03) | (0.03) |
| Change in GDP | 1.01 | 1.01 |
|  | (0.03) | (0.03) |
| Unemployment | 1.07*** | 1.07*** |
|  | (0.01) | (0.01) |
| Change in unemployment | 1.11*** | 1.11*** |
|  | (0.01) | (0.01) |
| Unemployment spending | 1.06 | 1.06 |
|  | (0.03) | (0.03) |
| ALMP Spending^a^ | 1.0003*** | 1.0003*** |
|  | (0.00001) | (0.00001) |
| Severance Payment Quartile | 0.96*** |  |
|  | (0.00) |  |
| Notice Period Quartile |  | 0.97*** |
|  |  | (0.00) |
| N | 348, 000 | 348, 000 |

*Notes:* a: spending on active labour market programs. Source: OECD.Stat (2015), P-values: * p<0.05 ; ** p<0.01; *** p<0.001. Standard errors in parentheses. Exponentiated coefficients show association of 1 unit increase in the dependent variable on the odds of reporting a health decline. To facilitate interpretation we transform the GDP, ALMP spending and unemployment variables as follows: coefficient of GDP shows association of 1000$ increase in GDP per capita; coefficient of ALMP spending shows association of $100 increase; coefficients of unemployment, change in unemployment and change in GDP show association of a 10% increase; coefficient of unemployment spending shows association of 100 euro increase.
